# Diagnostic, Prognostic, and Immunological Roles of HELLS in Pan-Cancer: A Bioinformatics Analysis

**DOI:** 10.3389/fimmu.2022.870726

**Published:** 2022-06-14

**Authors:** Xiao Liang, Linji Li, Yuchao Fan

**Affiliations:** ^1^ Department of Anesthesiology, West China Hospital, Sichuan University, Chengdu, China; ^2^ Department of Anesthesiology, Nanchong Central Hospital, The Second Clinical Medical College, North Sichuan Medical College, Nanchong, China; ^3^ Department of Anesthesiology, Sichuan Cancer Center, Sichuan Cancer Hospital & Institute, School of Medicine, University of Electronic Science and Technology of China, Chengdu, China

**Keywords:** HELLS, DNA repair, pan-cancer, tumor immunity, anti-tumour, bioinformatics

## Abstract

**Background:**

Inappropriate repair of DNA damage drives carcinogenesis. Lymphoid-specific helicase (HELLS) is an important component of the chromatin remodeling complex that helps repair DNA through various mechanisms such as DNA methylation, histone posttranslational modification, and nucleosome remodeling. Its role in human cancer initiation and progression has garnered recent attention. Our study aims to provide a more systematic and comprehensive understanding of the role of HELLS in the development and progression of multiple malignancies through analysis of HELLS in cancers.

**Methods:**

We explored the role of HELLS in cancers using The Cancer Genome Atlas (TCGA) and Genotype-Tissue Expression (GTEx) database. Multiple web platforms and software were used for data analysis, including R, Cytoscape, HPA, Archs4, TISIDB, cBioPortal, STRING, GSCALite, and CancerSEA.

**Results:**

High HELLS expression was found in a variety of cancers and differentially expressed across molecular and immune subtypes. HELLS was involved in many cancer pathways. Its expression positively correlated with Th2 and Tcm cells in most cancers. It also correlated with genetic markers of immunomodulators in various cancers.

**Conclusions:**

Our study elucidates the role HELLS plays in promotion, inhibition, and treatment of different cancers. HELLS is a potential cancer diagnostic and prognostic biomarker with immune, targeted, or cytotoxic therapeutic value. This work is a prerequisite to clinical validation and treatment of HELLS in cancers.

## Introduction

Exposure to toxic chemicals, oxidation, free radicals, ultraviolet light, and ionizing radiation damages human DNA, including double-stranded breaks (1, 2). Double-stranded breaks are difficult to repair (3). Faulty damage response signals and unrepaired DNA destabilize the genome, possibly leading to cancer (4).

Eukaryotic cells have evolved a set of mechanisms called the DNA damage response (DDR) to detect, signal, and repair DNA damage (4). The SWI/SNF2 superfamily of ATPases participates in this process by hydrolyzing ATP, which fuels chromatin remodeling (5, 6). Members of the SWI/SNF2 superfamily include SMARCA1 (SWI/snf-related, matrix-associated, actin-dependent regulatory chromatin group a) (SNF2L), SMARCA2 (BRM), SMARCA3 (LTF), SMARCA4 (BRG1), SMARCA5 (SNF2H), SMARCA6 (HELLS), SMARCAD1, and SMARCAL1. They belong to three different SWI/SNF2 subfamilies: SWI/SNF, ISWI, and INO80 (7). Previous works have studied the link between genetic susceptibility to cancers and mutational inactivation of SMARCA2 and SMARCA4 (8). However, SMARCA6 has attracted more attention in recent years.

SMARCA6, also known as HELLS (helicase, lymphoid-specific), LSH (lymphoid-specific helicase), or PASG (proliferation-associated SNF2-like gene), localizes on the human chromosome in the 10q23-q24 region of c3-d1 and belongs to the SWI2/SNF2 superfamily (9). HELLS plays a vital role in chromatin remodeling, DNA replication, repair, recombination, methylation, transcription regulation, and maintaining chromosomal stability (10). HELLS deletion causes perinatal death in mice, whereas HELLS mutation leads to embryonic multi-organ and stem cell defects (11, 12).

How HELLS affects cancer development and progression is important because chromosomal instability contributes to cancer. The role of HELLS in cancer has received a lot of attention from researchers in the past years, and aberrant HELLS expression is reportedly involved in the development of a variety of malignancies and is linked to poor prognosis in these tumors (13–16). HELLS has a broad and diverse regulatory role in cancer and is closely linked to well-known molecules or pathways regulating cancer (17) which have important implications for cancer therapy. This suggests that HELLS is an attractive target for tumor diagnosis and treatment. However, no studies have yet analyzed the role of HELLS in pan-cancer.

Due to the rapid development of biological databases in recent years, we have been able to access large samples of bioinformatics data on a wide range of cancer and associated normal tissues in publicly available databases. This makes the results of bioinformatics analyses with large sample sizes or incorporating a large number of cancer types extensive or representative. It is also possible to find other important researchable directions beyond the previously reported results. Here we present a comprehensive bioinformatic analysis of HELLS expression, diagnostic value, prognostic value, functional enrichment, and immune infiltration in pan-cancer. We found that HELLS is valuable in the diagnosis and prognosis of a wide range of cancer and regulates cancer by a variety of mechanisms. The role of HELLS in some of these cancers has not been reported and there are also previously unexplored mechanisms or pathways that regulate progression in the cancers where HELLS has been reported. The relationship between HELLS and tumor-infiltrating lymphocytes, immunostimulators, immunoinhibitors, MHC molecules, chemokines, and chemokine receptors in 33 cancers has also rarely been reported in previous studies. This may contribute to the understanding of the role of HELLS in cancer immunomodulation and immunotherapy.

## Materials and Methods

### HELLS Expression and Datasets Obtained

We searched HPA (https://www.proteinatlas.org/) and got the HELLS RNA and protein expression summary in humans. Details of HELLS expression in tissues and cell lines were obtained from the Harmonizome database (https://maayanlab.cloud/Harmonizome/). TCGA (https://cancergenome.nih.gov) and GTEx (https://gtexportal.org/) were used to obtain the HELLS mRNA expression of tumor samples, corresponding paracancer samples, and normal samples. The 33 cancer types included: Adrenocortical carcinoma (ACC), Bladder Urothelial Carcinoma (BLCA), Breast invasive carcinoma (BRCA), Cervical squamous cell carcinoma and endocervical adenocarcinoma (CESC), Cholangiocarcinoma (CHOL), Colon adenocarcinoma (COAD), Lymphoid Neoplasm Diffuse Large B-cell Lymphoma (DLBC), Esophageal carcinoma (ESCA), Glioblastoma multiforme (GBM), Head and Neck squamous cell carcinoma (HNSC), Kidney Chromophobe (KICH), Kidney renal clear cell carcinoma (KIRC), Kidney renal papillary cell carcinoma (KIRP), Acute Myeloid Leukemia (LAML), Brain Lower Grade Glioma (LGG), Liver hepatocellular carcinoma (LIHC), Lung adenocarcinoma (LUAD), Lung squamous cell carcinoma (LUSC), Mesothelioma (MESO), Ovarian serous cystadenocarcinoma (OV), Pancreatic adenocarcinoma (PAAD), Pheochromocytoma and Paraganglioma (PCPG), Prostate adenocarcinoma (PRAD), Rectum adenocarcinoma (READ), Sarcoma (SARC), Skin Cutaneous Melanoma (SKCM), Stomach adenocarcinoma (STAD), Testicular Germ Cell Tumors (TGCT), Thyroid carcinoma (THCA), Thymoma (THYM), Uterine Corpus Endometrial Carcinoma (UCEC), Uterine Carcinosarcoma (UCS), Uveal Melanoma (UVM).

We excluded the samples with “0” values for gene expression. Paired samples are retained for paired sample analysis. RNA-sequencing data in Fragments Per Kilobase per Million format were converted and normalized by the Toil process as transcripts per million reads and log2 transformed for further analysis.

R (v 3.6.3) software was used to perform statistical analyses in this study. The “ggplot2” (v3.3.3) package was used to present HELLS gene expression as bar graph in patients with the 33 cancers. The median method of gene expression was selected for cutoff values. The Wilcoxon rank-sum test was performed to determine the difference between groups.

### Receiver Operator Characteristic (ROC) Curve of HELLS in the 33 Cancers

ROC curves were used to estimate the diagnostic value of HELLS in the 33 cancers. The data used to construct ROC curves are derived from the mRNA expression of HELLS in cancer and corresponding normal tissues in TCGA and GTEx. ROC curves were calculated by package “pROC” (v1.17.0.1) of R software and plotted by package “ggplot2”. The Area under Curve (AUC), cutoff, sensitivity, specificity, positive predictive value, negative predictive value, and Youden’s index (YI) were also calculated. The closer the AUC is to 1, the better the diagnosis is. 0.5 to 0.7 AUC has low accuracy, 0.7 to 0.9 AUC has good accuracy, and 0.9 or more AUC has high accuracy. YI indicates the total ability of the screening method to detect real patients versus non-patients. The larger the index, the more valid the screening method.

### Survival Analysis of HELLS in the 33 Cancers

The “survival” package was used to conduct Kaplan–Meier (K-M) analysis. The overall survival (OS), disease-specific survival (DSS), and progress-free interval (PFI) rates between the high- and low- HELLS gene expression groups were compared in the 33 cancers. The p-value was determined by Cox regression analysis. The forest plots plotted the hazard ratio (HR), 95% confidence interval and p-value of survival curves were calculated and visualized by “survminer” and “ggplot2” package.

### HELLS Expression in Different Molecular and Immune Subtypes of Cancers

For investigating the associations between HELLS expression and molecular or immune subtypes in the 33 cancers, the “subtype” module of TISIDB database was performed. The TISIDB database combines various data types to evaluate cancer and immune system interaction. HELLS mRNA expression was investigated in different immune subtypes, including C1 (wound healing), C2 (IFN-γ dominant), C3 (inflammatory), C4 (lymphocyte deplete), C5 (immunologically quiet), and C6 (TGF-β dominant).

### Genetic Alteration Analysis of HELLS

The cBioPortal (https://www.cbioportal.org/) was searched for genetic alteration information of HELLS. All the TCGA PanCancer Atlas Studies were included. Somatic mutation frequency and genomic information of HELLS mutation in cancers were explored with the “cancer types summary and mutations” and “mRNA vs. study” module. The mutations sites were obtained from “mutations” modules.

### Protein-Protein Interaction (PPI) Network Analyses of HELLS

STRING database (https://string-db.org/) was used to collect and integrate potential protein interactions with HELLS. The relevant genes obtained were used to conduct a PPI network analysis. A confidence score > 0.7 was set as the significance threshold. Then we imported relevant data into Cytoscape (v3.8.2) for visualization and subsequent analysis. The cytoHubba plugins of Cytoscape were used to identify key modules, and the top 10 nodes, ranked using MCC of cytoHubba, are presented as hub genes. The pathlinker plugins were performed to reconstruct signaling pathways from the top 10 hub genes. Pathlinker (18) can efficiently calculate multiple short paths from receptors to transcription factors (TFs) in a pathway from the PPI network. We have subsequently explored correlations between hub genes in a variety of cancers as well.

### Functional Enrichment Analysis of HELLS

We conduct Gene Ontology (GO) function and Kyoto Encyclopedia of Genes and Genomes (KEGG) enrichment analyses for the gene closely interact with HELLS which were obtained from STRING *via* the “clusterProfiler” and “org.Hs.eg.db” packages of R. The cutoff threshold was set as p-value < 0.01 for GO and KEGG pathway enrichment analyses. The results were presented as a bubble chart *via* the “ggplot2.”

### GSCALite

GSCALite (19) is a genomic cancer analysis platform that integrates cancer genomic data from TCGA for the 33 cancer types, drug response data from GDSC, CTRP, and normal tissue data from GTEx in a unified data analysis process for gene set analysis. We explored the famous cancer-related pathways activated or inhibited in the 33 cancer types of HELLS on this platform. The pathways include TSC/mTOR, RTK, RAS/MAPK, PI3K/AKT, Hormone ER, Hormone AR, epithelial–mesenchymal transition (EMT), DDR, Cell Cycle, and Apoptosis pathways.

### Gene Set Enrichment Analysis

The “clusterProfiler” package performed Gene set enrichment analysis (GSEA) to determine the biological pathway differences between high- and low-HELLS groups. Remarkably changed pathways were considered as a false discovery rate (FDR) < 0.25 and an adjusted p-value < 0.05. Each analysis should perform the Gene set permutation 1,000 times. The top 15 entries of the enrichment results are presented as mountain maps. The “ggplot2” package in R was used to visualize the results of GSEA.

### CancerSEA

We have studied the functional status of HELLS in a variety of cancers using CancerSEA (20) which is a database capable of studying the functional status of cancer cells at the single-cell level. The average correlation between HELLS and functional states in 18 cancers was explored, including invasion, metastasis, proliferation, epithelial–mesenchymal transition (EMT), angiogenesis, apoptosis, cell cycle, differentiation, DNA damage, DNA repair, hypoxia, inflammation, quiescence, and stemness. The threshold for correlation between HELLS and cancer functional states was set at a correlation strength of 0.3 and a *p*-value of less than 0.05.

### Immunogenomic Analyses of HELLS in the 33 Cancers

The “GSVA” package with “ssGSEA” algorithm was used to explore the correlation between the HELLS expression and tumor-infiltrating lymphocytes, immunostimulators, immunoinhibitors, MHC molecules, chemokines, and chemokine receptors in the 33 cancers. The correlation was evaluated *via* Spearman’s correlation and p-values < 0.05 were considered statistically significant. The “ggplot2” package was performed to visualize the correlations as heatmaps.

## Results

### Expression Landscape and Pan-Cancer Expression of HELLS

**Figure 1:**
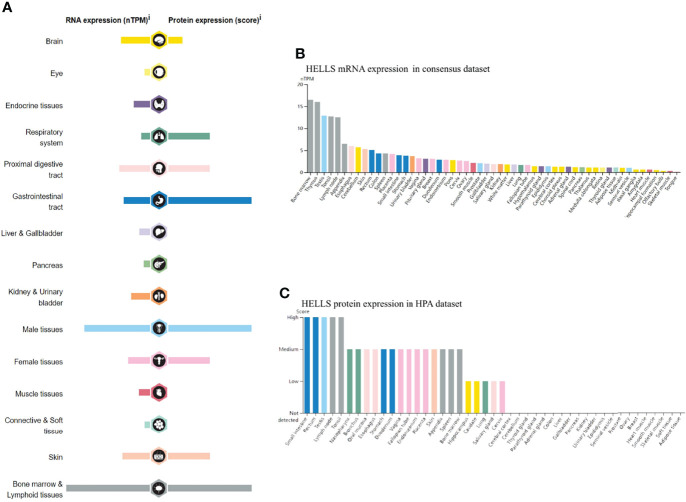
RNA and protein expression profile of HELLS in human organs and tissues. **(A)** The summary of HELLS mRNA and protein expression in human organs and tissues; **(B)** HELLS mRNA expression summary in different human organs and tissues based on consensus dataset; **(C)** HELLS protein expression summary in different human organs and tissues.

The mRNA and protein of HELLS are widely expressed in various organs and tissues (**Figure 1A**). The result obtained from the consensus dataset, which included 375 normal tissues in HPA database and 13,084 samples in GTEx, showed mRNA of HELLS expressed primarily in bone marrow, thymus, testis, tonsil, lymph node, appendix, esophagus, cerebellum, skin, and rectum (**Figure 1B**). The protein of HELLS data was acquired from the HPA database which has 144 individuals corresponding to 44 samples of different normal tissue types. The protein of HELLS is mainly expressed in the small intestine, rectum, testis, lymph node, tonsil, nasopharynx, bronchus, oral mucosa, esophagus, and stomach. (**Figure 1C**). The details of HELLS mRNA expression in different tissues and cell lines are shown in **Supplemental Figure 1**.

**Figure 2:**
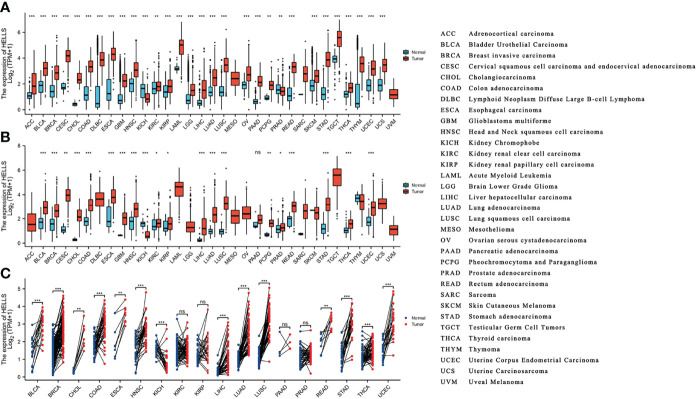
The expression of HELLS mRNA in pan-cancer. **(A)** Expression of HELLS between the 33 cancers and normal tissues in unpaired sample analysis; **(B)** Expression of HELLS between the 33 cancers and paracancerous tissues in unpaired sample analysis; **(C)** Paired sample analysis of HELLS mRNA expression between 18 cancers and paracancerous tissues in BLCA, BRCA, CHOL, COAD, ESCA, HNSC, KICH, KIRC, KIRP, LIHC, LUAD, LUSC, PAAD, PRAD, READ, STAD, THCA and UCEC. ^∗^ *p* < 0:05, ^∗∗^ *p* < 0:01, ^∗∗∗^ *p* < 0:001. ns, Not Significant.

The HELLS mRNA expression was evaluated in the 33 cancer types. As **Figure 2A** shows, 15,776 samples were included in the unpaired sample analysis, compared with normal samples low HELLS mRNA expression was observed in PRAD (*p* = 0.002) and high HELLS mRNA expression was observed in ACC, BLCA, BRCA, CESC, CHOL, COAD, DLBC, ESCA, GBM, HNSC, KICH, KIRP, LAML, LGG, LIHC, LUAD, LUSC, OV, PAAD, READ, SKCM, STAD, TGCT, THCA, THYM, UCEC, UCS (all *p* < 0.001), KIRC, and PCPG (*p* = 0.007). MESO, SARC, and UVM could not be analyzed due to the lack of sufficient normal samples. Compared to paracancerous tissue, HELLS mRNA expressed significantly lower in KICH (*p* < 0.001) and significantly higher in BLCA, BRCA, CHOL, COAD, ESCA, GBM, HNSC, LUAD, LUSC, PRAD *(p* = 0.045), READ, STAD, THCA, THYM, UCEC (all *p* < 0.001) and CESC (*p* = 0.003), KIRC (*p* = 0.011), KIRP (*p* = 0.031), PCPG (*p* = 0.006). This paired analysis included 10,534 samples (**Figure 2B**). ACC, DLBC, LAML, LGG, MESO, OV, SARC, SKCM, TGCT, THYM, UCS, and UVM could not be analyzed due to the lack of sufficient paracancerous samples. There was no difference shown in PAAD (*p* > 0.05). Among the paired sample analyses that was performed with 730 samples in 18 cancers and 730 paracancerous samples, HELLS mRNA expression was increased in BLCA, BRCA, COAD, HNSC, KICH, LIHC, LUAD, LUSC, STAD, UCEC (all *p* < 0.001) and CHOL (*p* = 0.004), ESCA (*p* = 0.008), and READ (*p* = 0.004). It was decreased in KICH (*p* = 0.001) (**Figure 2C**).

### The Diagnostic Value of HELLS in the 33 Cancers

**Figure 3:**
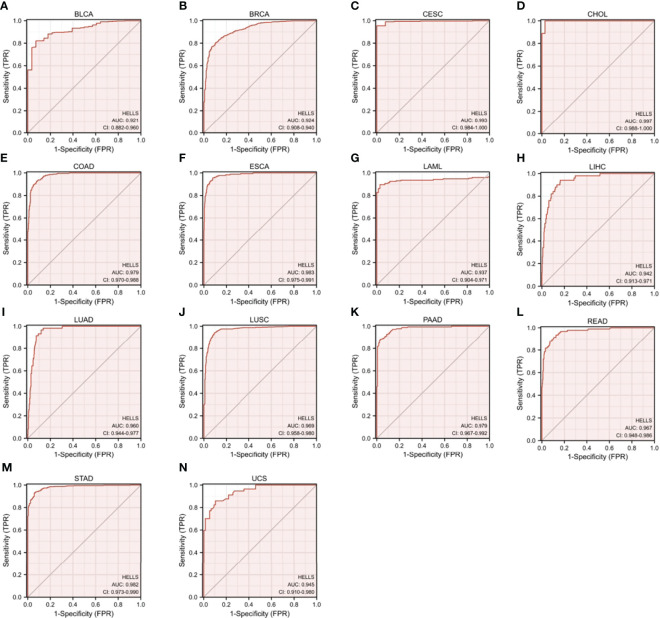
Receiver Operator Characteristic (ROC) curve of HELLS in 14 Cancers. Cancers with AUC > 0.9 for HELLS: **(A)** BLCA, **(B)** BRCA, **(C)** CESC, **(D)** CHOL, **(E)** COAD, **(F)** ESCA, **(G)** LAML, **(H)** LICH, **(I)** LUAD, **(J)** LUSC, **(K)** PAAD, **(L)** READ, **(M)** STAD, **(N)** UCS.

As shown in **Figures 3A–N**, HELLS has a good diagnostic value in a variety of cancers. Its AUC was greater than 0.7 in 27 cancers and even exceeded 0.9 in 14 cancers including BLCA (AUC = 0.921), BRCA (AUC = 0.924), CESC (AUC = 0.993), CHOL (AUC = 0.997), COAD (AUC = 0.979), ESCA (AUC = 0.983), LAML (AUC = 0.937), LICH (AUC = 0.942), LUAD (AUC = 0.960), LUSC (AUC = 0.969), PAAD (AUC = 0.979), READ (AUC = 0.967), STAD (AUC = 0.982) and UCS (AUC = 0.945) (**Supplemental Table 1**), which had high diagnostic value.

### Survival Analysis of HELLS in the 33 Cancers

**Figure 4:**
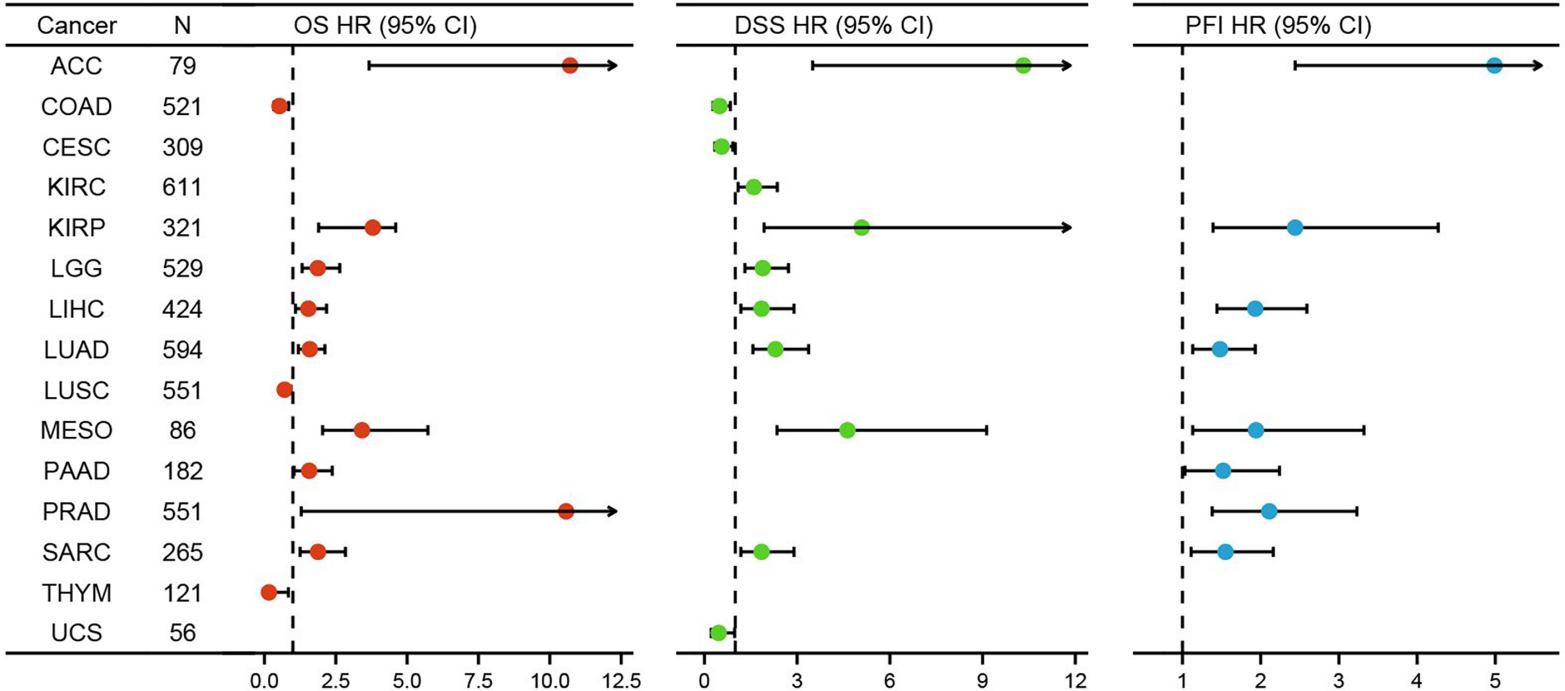
K-M analysis for high- and low- HELLS gene expression in cancers. Forest plot of HELLS OS in 12 cancers (Red), DSS in 11 cancers (Green), and PFI in 8 cancers (Blue).

**Figure 5:**
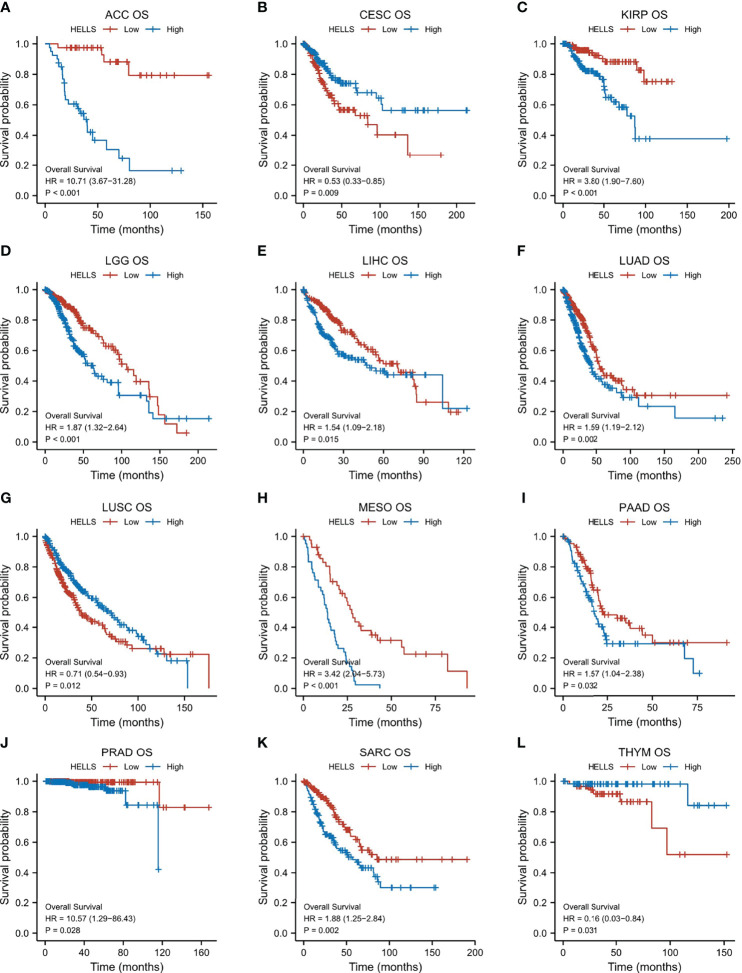
Correlations between HELLS and prognosis in 12 cancers. OS K-M curve for HELLS in 12 cancers. The unit of X-axis is month. **(A)** ACC, **(B)** CESC, **(C)** KIRP, **(D)** LGG, **(E)** LIHC, **(F)** LUAD, **(G)** LUSC, **(H)** MESO, **(I)** PAAD, **(J)** PRAD, **(K)** SARC, **(L)** THYM.

For purpose of evaluating the prognosis value of HELLS, we carried out the Kaplan–Meier analysis. Cox regression analysis of the 33 cancers showed that HELLS expression in 12 cancers was significantly associated with OS (**Figure 4**, **Supplemental Table 2**). The result found that high HELLS groups have statistically better OS than those for the low HELLS groups in CESC, LUSC, and THYM. However, the low HELLS groups show statistically better OS than high HELLS groups in ACC, KIRP, LGG, LIHC, LUAD, MESO, PAAD, PRAD, and SARC (**Figures 5A–L**). Regarding DSS of the 33 cancers, the HELLS play a protective role for CESC, COAD, and UCS and a risk role for ACC, KIRC, KIRP, LGG, LIHC, LUAD, MESO, and SARC (**Figure 4**, **Supplemental Figures 2A–K**, **Supplemental Table 2**). For PFI analysis in the 33 cancers, the HELLS play a risk role for ACC, KIRP, LIHC, LUAD, MESO, PAAD, PRAD, and SARC (**Figure 4**, **Supplemental Figures 3A–H**, **Supplemental Table 2**).

### HELLS Expression in Different Immune and Molecular Subtypes of the 33 Cancers

**Figure 6:**
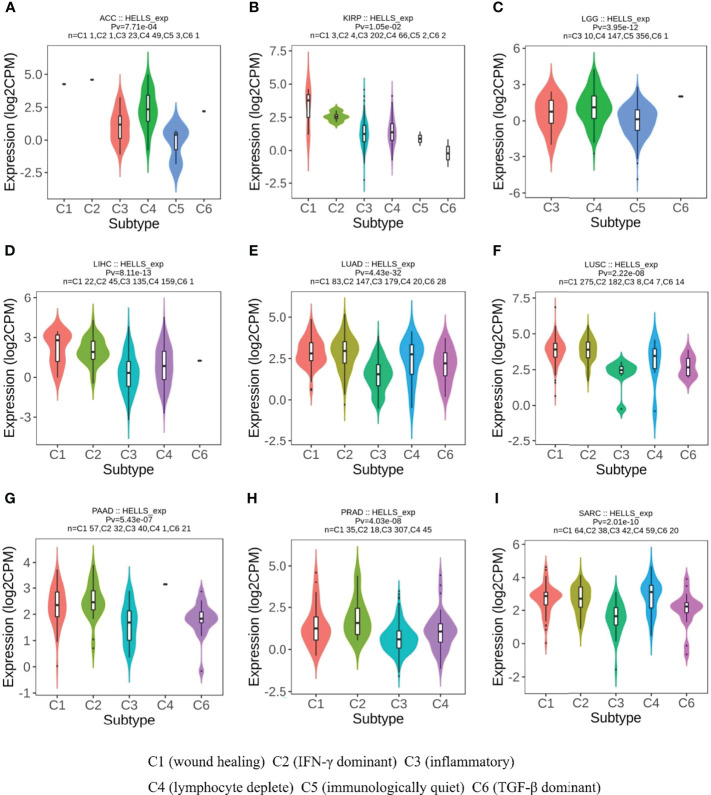
Correlations between HELLS expression and immune subtypes in 9 cancers. **(A)** ACC, **(B)** KIRP, **(C)** LGG, **(D)** LIHC, **(E)** LUAD, **(F)** LUSC, **(G)** PAAD, **(H)** PRAD, **(I)** SARC. C1 (wound healing), C2 (IFN-γ dominant), C3 (inflammatory), C4 (lymphocyte deplete), C5 (immunologically quiet), and C6 (TGF-β dominant).

**Figure 7:**
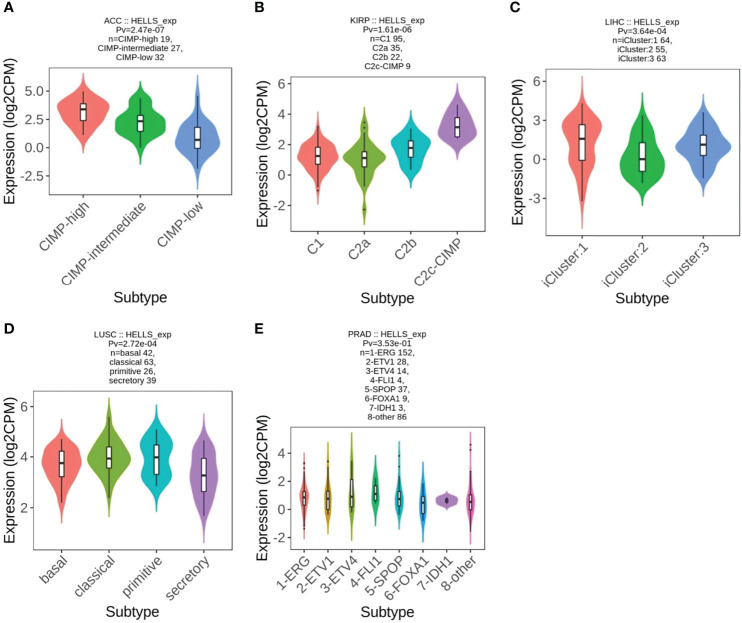
Correlations between HELLS expression and molecular subtypes in 5 cancers. **(A)** ACC, **(B)** KIRP, **(C)** LIHC, **(D)** LUSC, **(E)** PRAD.

From the previous results, we found that the high or low level of HELLS expression had an impact on the OS of 12 cancers. We therefore analyzed HELLS expression in the immune and molecular subtypes of these cancers and 21 other cancers. The results show that HELLS expresses significantly differently in nine of 12 cancers for immune subtypes, including ACC (six subtypes), KIRP (six subtypes), LGG (four subtypes), LIHC (five subtypes), LUAD (five subtypes), LUSC (five subtypes), PAAD (five subtypes), PRAD (four subtypes), and SARC (five subtypes) (**Figures 6A–I**). For molecular subtypes, HELLS expresses significantly differently in five cancer types, including ACC, KIRP, LIHC, LUSC and PRAD (**Figures 7A–E**). For the other 21 cancers, significant differential expression of HELLS is observed in the immune subtypes of BRCA, COAD, ESCA, HNSC, KICH, KIRC, OV, PCPG, READ, SKCM, STAD, TGCT, THCA and UCEC (**Supplemental Figures 4A–N**) and the molecular subtypes of BRCA, COAD, HNSC, OV, PCPG, SKCM, STAD, UCEC (**Supplemental Figures 5A–H**).

### Genetic Alteration of HELLS

**Figure 8:**
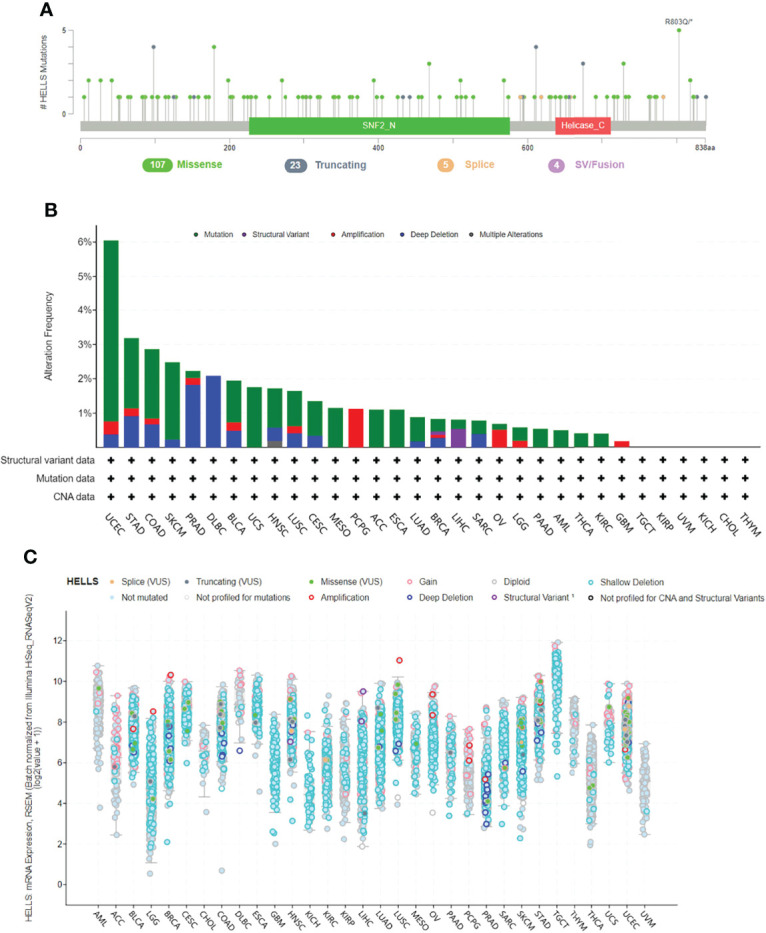
Genetic alteration of HELLS in 32 cancers. **(A)** Mutation diagram of HELLS across protein domains; **(B)** Bar chart of HELLS mutations in 32 cancer studies based on TCGA PanCancer Atlas Studies; **(C)** Mutation counts and types of HELLS in 32 cancers.

The genetic mutations of HELLS expression in cancers were analyzed through the cBioPortal online tool. All the TCGA PanCancer Atlas Studies with 32 studies and 10,967 samples were included. We found 139 mutation sites between amino acids 0 and 838, including 107 missense mutations, 23 truncating, five splices, four SV/fusion, and R803Q as the most frequent mutation site (**Figure 8A**). The most predominant mutation types were Missense mutation, Amplification, and Deep Deletion. HELLS mutations were most commonly seen in UCEC, STAD, COAD, SKCM, PRAD, DLBC, BLCA, and UCS (**Figure 8B**). Among the 32 cancers, shallow deletion was common in the expression of HELLS mRNA in all cancers except AML, ACC, DLBC, PCPG, THYM, THCA, and UVM (**Figure 8C**).

### The PPI, Functional Enrichment, and Gene Set Enrichment of HELLS in Cancers

**Figure 9:**
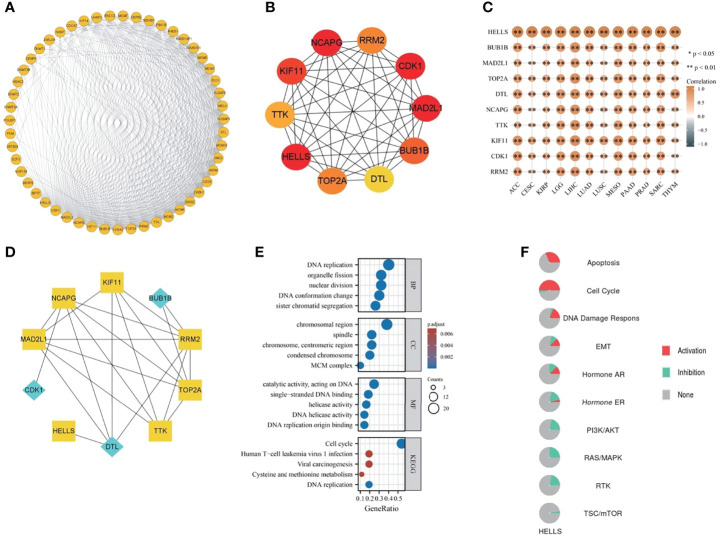
The PPI network and functional enrichment analysis of HELLS. **(A)** The PPI network of HELLS, **(B)** The top ten hub genes of PPI network, **(C)** The association hub gene with HELLS in 12 cancers present as heatmap. ^∗^ *p* < 0:05, ^∗∗^ *p* < 0:01, **(D)** The signaling pathway reconstruct form hub genes. Blue diamonds represent receptors and yellow squares represent transcription factors in the signaling pathway, **(E)** GO/KEGG pathway enrichment for HELLS and closed interact genes, **(F)** HELLS with pathway activity or inhibition.

A total of 50 genes, closely linked to HELLS, were obtained from string and a PPI network was constructed by searching at the given threshold (**Figure 9A**). The top 10 hub genes were BUB1B, MAD2L1, TOP2A, DTL, NCAPG, TTK, KIF11, CDK1, HELLS, and RRM2 (**Figure 9B**). The top 10 hub genes are all closely linked in cancers in which HELLS expression affects prognosis (all *p* < 0.001) (**Figure 9C**). In the top 10 hub genes, BUB1B, DTL, and CDK1 were receptors and the remains were TFs (**Figure 9D**). GO/KEGG enrichment analyses were performed on these genes. The RNA functional included three categories: the biological process (BP), molecular function (MF), and cellular component (CC). The top GO terms of BP were DNA replication, sister chromatid segregation, DNA conformation change, nuclear division, and organelle fission; CC were chromosomal region, chromosome, centromeric region, MCM complex, condensed chromosome, spindle and MF were DNA replication origin binding, catalytic activity, acting on DNA, single-stranded DNA binding, DNA helicase activity, and helicase activity. The top KEGG pathways were cell cycle, DNA replication, viral carcinogenesis, cysteine and methionine metabolism, and human T-cell leukemia virus I infection (**Figure 9E**). The pathways activated by HELLS, mainly in cancers, were apoptosis, cell cycle, DDR, EMT, and hormone AR, and those inhibited are hormone AR, RAS/MAPK, PI3K/AKT, RTK, and TSC/mTOR (**Figure 9F**).

**Figure 10:**
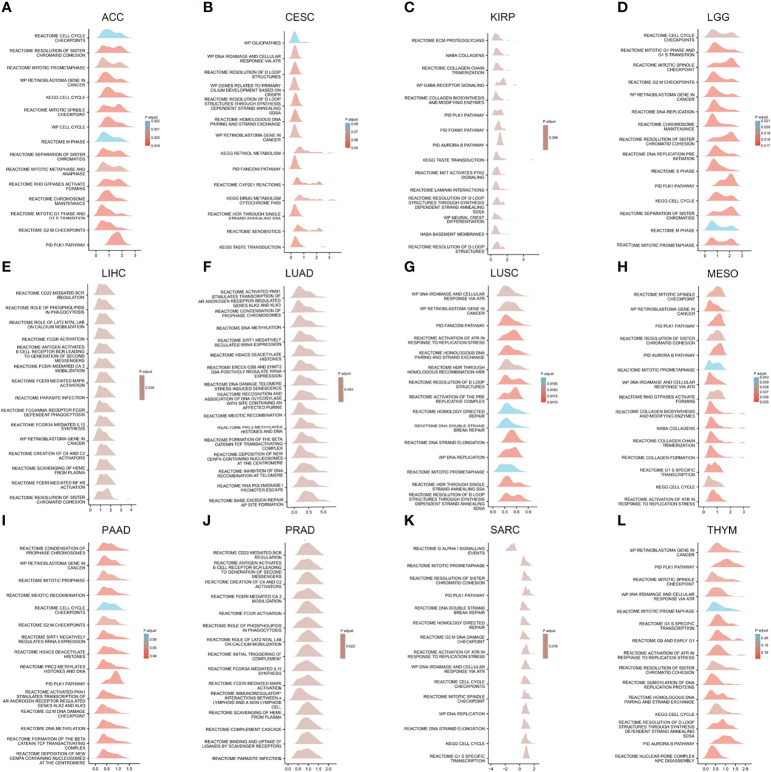
GSEA functional enrichment analysis of HELLS expression in 12 cancers. The top 15 GSEA functional enrichment pathways of HELLS in **(A)** ACC, **(B)** CESC, **(C)** KIRP, **(D)** LGG, **(E)** LIHC, **(F)** LUAD, **(G)** LUSC, **(H)** MESO, **(I)** PAAD, **(J)** PRAD, **(K)** SARC, **(L)** THYM. The Y-axis represents one gene set and the X-axis is the distribution of logFC corresponding to the core molecules in each gene set.

The GSEA results of 12 cancers are shown in **Figures 10A–L**. Common enrichment pathways are cell cycle checkpoints, resolution of sister chromatid cohesion, mitotic prometaphase, retinoblastoma gene in cancer, cell cycle, mitotic spindle checkpoint, M phase, DNA irdamage and cellular response via ATR. resolution of D loop structures, related to primary cilium development based on CRISPR and so on. These results suggest that in a variety of cancers, HELLS is closely associated with the processes of DNA strand replication, repair, recombination, and transcription.

### Functional States of HELLS in scRNA-Seq Datasets

**Figure 11:**
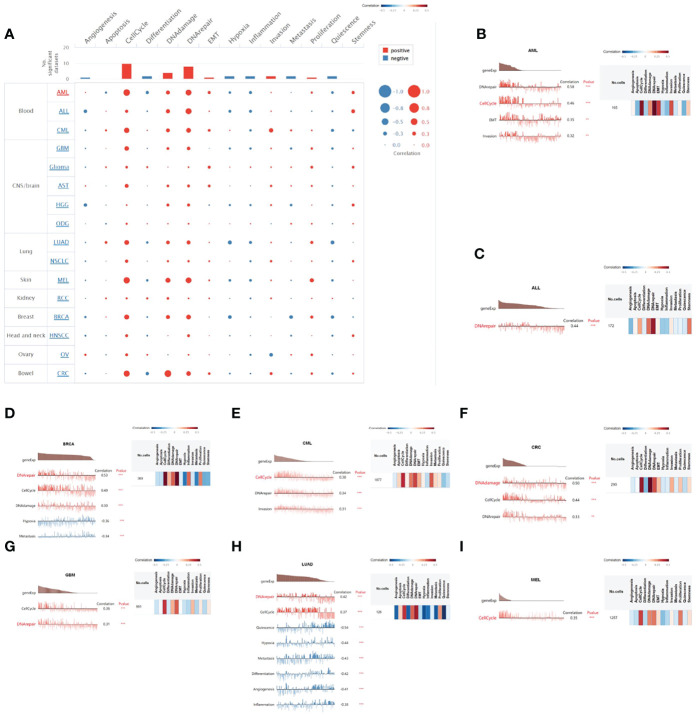
The correlation of HELLS with functional state in cancers. **(A)** The interactive bubble chart present correlation of HELLS with functional state in 16 cancers. The correlation of HELLS with functional state in **(B)** AML, **(C)** ALL, **(D)** BRCA, **(E)** CML, **(F)** CRC, **(G)** GBM, **(H)** LUAD, **(I)** MEL. X-axis represents different gene sets; ***p < 0.001, **p < 0.01, *p < 0.05.

We explored the functional state of HELLS in various cancer types *via* the CancerSEA, which can enable us to analyze the correlation of HELLS with multiple functional states of cancer cells at the single-cell level. The results showed that HELLS expression had a positive correlation with the cell cycle, DNA damage, DNA repair, invasion, and proliferation. Negative correlations were observed between HELLS expression and angiogenesis, hypoxia, inflammation, and quiescence but these negative correlations were relatively weak (**Figure 11A**). We then explored the correlation between HELLS and the functional status in specific cancers. The results found that HELLS positively correlated with DNA repair, cell cycle, EMT, and invasion in acute myelogenous leukemia (AML); with DNA repair in acute lymphoblastic leukemia; with DNA repair, cell cycle, and DNA damage in BRCA; with cell cycle, DNA repair, and invasion in chronic myelogenous leukemia; with DNA damage, cell cycle, and DNA repair in colorectal cancer; with cell cycle and DNA repair in GBM; with DNA repair and cell cycle in LUAD; and with cell cycle in melanoma (MEL). Conversely, the HELLS were negatively correlated with hypoxia and metastasis in BRCA, with quiescence, hypoxia, metastasis, differentiation angiogenesis, and inflammation in LUAD (**Figures 11B–I**).

### Immunogenomic Analyses of HELLS in the 33 Cancers

**Figure 12:**
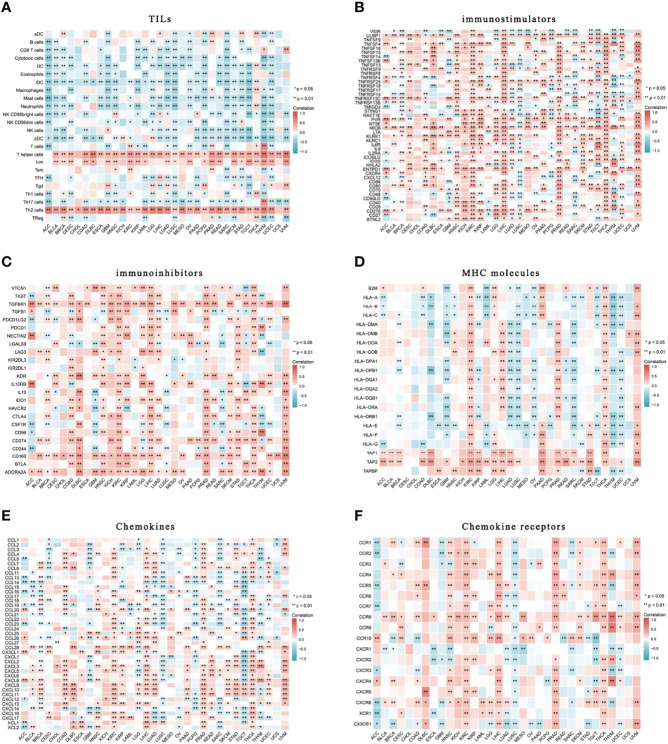
Correlation of HELLS with TILs and immunoregulation-related genes in 33 cancers. Correlations between HELLS expression and **(A)** TILs, **(B)** Immunostimulators, **(C)** Immunoinhibitors, **(D)** MHC molecules, **(E)** Chemokines, **(F)** Chemokine receptors. ^∗^ *p* < 0:05, ^∗∗^ *p* < 0:01.

To assess the relationship of HELLS with immune infiltration and immune regulation, we constructed a heat map of HELLS with markers of immune cells or factors. Our results showed that HELLS was positively correlated with the level of central memory T cell(Tcm), T helper, and Th2 infiltration in the 33 cancers and negatively correlated with the level of infiltration of most other immune cells (**Figure 12A**). In terms of immunostimulators, HELLS was positively correlated with most immunostimulators in the 33 cancers, especially in several HNSC, KIRC, KIRP, LIHC, PRAD, THCA, and UVM (**Figure 12B**). Interestingly, HELLS also showed a positive correlation with most immunoinhibitors, particularly in DLBC, HNSC, KIRC, KIRP, LIHC, PRAD, STAD, THCA, and UVM (**Figure 12C**). HELLS showed a positive correlation with most MHCs in KIRC, LGG, LIHC, PAAD, PRAD, THCA, UVM and a negative correlation with most MHCs in GBM, LUAD, LUSC, OV, SARC, THYM, and UCEC (**Figure 12D**). The majority of cytokines in COAD, KIRC, LGG, LIHC, PRAD, THCA, and UVM showed a positive correlation with HELLS, while in GBM, LUSC, PCPG, SARC, and TGCT there were negative correlations (**Figure 12E**). As for cytokine receptors, HELLS showed a positive correlation with most of them in HNSC, KIRC, LIHC, PRAD, THCA, and a negative correlation in LUSC (**Figure 12F**).

## Discussion

HELLS plays a major role in epistasis regulation and DNA repair. This member of the SN2 family of chromatin remodeling proteins has earned attention for its involvement in tumor development, progression, and treatment. Our bioinformatics approach revealed the role HELLS plays in pan-cancer. Here, we determined the expression levels of HELLS mRNA and protein in human organs, tissues, and cell lines and compared them with those found in various cancers. We then quantified the diagnostic and prognostic values of HELLS expression in various cancers and differentiated its expression levels across several immune and cellular subtypes of cancers. The most common types of HELLS mutations and their locations were also identified. PPI and miRNA regulatory networks were constructed for HELLS, its activation or inhibition in famous pathways investigated, its functionally enriched pathways revealed, and its activation or inhibition at the single-cell level in specific cancers explored. Finally, we correlated HELLS expression levels with the infiltration levels of immune cells and regulators in various cancers.

Although HELLS is not widely expressed in all normal tissues, our results from TCGA and GTEx show that its expression was significantly higher in most cancers than in normal tissues. The AUC of the ROC exceeded 0.7 in 27 cancers and 0.9 in 14 cancers, suggesting that increased HELLS expression contributes to the development of many cancers and can be a new diagnostic marker in clinical practice. Our results show an AUC of 0.865 in the ROC of HNSC. Previous studies (21, 22) showed that the key oncogene FOXM1 promoted the progression of HNSC and OSCC through the downstream target HELLS. A combination of HELLS and FOXM1 may serve as a biomarker for early cancer detection, malignant transformation, and progression in HNSC and OSCC. HELLS expression was also elevated in breast cancer (23), non-smallcell lung cancer (15), and liver cancer (24). Kim et al. (25) showed that HELLS mRNA levels in the blood of patients with distant organ metastases from malignant melanoma were significantly higher than those of localized patients. In this study, the area under the ROC curve of HELLS was greater than that of serum LDH, which is currently one of the most useful serum prognostic indicators for metastatic melanoma. This suggests that HELLS mRNA can be a serum biomarker for metastasis in some cancers.

Our study used K-M analysis to evaluate the prognostic value of HELLS in the 33 cancers. Concerning OS, HELLS was a risk factor in several cancers, including ACC, KIRP, LGG, LIHC, LUAD, MESO, PAAD, PRAD, and SARC, but was a protective factor in cancers such as CESC and LUSC. We also performed DSS and PFI analysis of HELLS in the 33 cancers because other factors may confound OS, and deaths from noncancerous causes do not fully represent the impact of cancer progression on survival. High HELLS showed as a risk factor in ACC, KIRP, LIHC, LUAD, MESO, and SARC for OS, DSS, and PFI, in PAAD and PRAD for OS and PFI. It showed as a risk factor in LGG but a protective factor in COAD for OS and DSS. This suggests that HELLS has a better prognostic predictive value in these cancers. Previous studies have associated HELLS with poor prognosis in various cancers. For example, HELLS can promote the progression of nasopharyngeal carcinoma by regulating fumarate hydratase expression (25). HELLS overexpression can also promote the growth of hepatocellular carcinoma by activating the transcription of centromere protein F (24). In glioma, HELLS regulates the proliferation of stem cell-like glioblastoma stem cells by interacting with key oncogenic TFs E2F3 and MYC; targeted the inhibition of HELLS and prolonged survival in mice and improved the prognosis of patients with increased HELLS expression (17). This suggests that HELLS is not only a predictor of tumor prognosis but also a possible target for tumor therapy. Targeting HELLS could complement chemotherapeutic agents. Xuyang Hou et al. (26) found that HELLS was highly expressed in patients with prostate cancer and greater expression was linked to more advanced clinical stages and worse prognosis. HELLS also promotes the development of pancreatic cancer through epigenetic silencing of the tumor suppressor TGFBR3, whereas inhibition of HELLS slows tumor growth and increases sensitivity to the chemotherapeutic agent cisplatin.

HELLS expression varied across different molecular or immune subtypes of cancer. These types of cancer were not limited to those where survival is determined by HELLS expression. HELLS is expressed abnormally in a particular subtype of cancer, which results in outcomes that may not be reflected in the overall population of that cancer. This could explain why differential HELLS expression does not affect survival in certain cancers, whereas its varied expression in different molecular or immune subtypes plays a different role in the prognosis of various cancers. Follow-up studies of HELLS expression in cancer will require inclusion of various subtypes and subtype-specific groups and targeting of unique molecular or immunological subtypes.

We observed that HELLS was mutated infrequently in 32 cancers and 6% of the UCEC population had HELLS mutations, which was the highest of all cancer types. Although Deep deletion of HELLS was present in UCEC, STAD, COAD, SKCM, PRAD, DLBC, HNSC, LUSC, CESC, LUAD, BRCA, and SARC, HELLS expression was higher than normal tissue in all the cancers except in PRAD, where HELLS expression was lower than normal tissues. This may be due to the fact that although the overall frequency of HELLS mutations in cancer is not high, we can see that the majority of HELLS mutations in PRAD are Deep deletion, and the number of samples with Deep deletion in HELLS mRNA expression is highest in PRAD compared to other cancers. In addition, the mutation types of HELLS in PCPG were almost exclusively amplified, which is consistent with the fact that the mutation types of HELLS mRNA expressed on PCPG samples were mainly Gain and amplification.

A PPI network was created to further elucidate the biological function of HELLS. We identified ten hub genes and explored how they correlated with HELLS expression in various cancers. HELLS expression was closely associated with these hub genes, suggesting their complementary biological roles in cancer. Among these hub genes, MAD2L1, TOP2A, NCAPG, TTK, KIF11, HELLS, and RRM2 may affect biological changes after receiving second messengers from activated BUB1B, DTL, and CDK1. BUB1B encodes a kinase involved in spindle checkpoint function, whose impairment is associated with various cancers (27–30). CDK1 encodes the catalytic subunit of a highly conserved protein kinase complex that is required for the G1/S and G2/M phase transitions of the eukaryotic cell cycle. Moreover, CDK1 regulates many cancers through multiple signaling pathways (31–34). DTL is also associated with poor prognosis in various cancers (35–37). Our GSEA results showed that the top pathways enriched in ACC, LGG, MESO, and THYM contained mitotic spindle checkpoints, whereas the top pathways enriched in ACC, MESO, PAAD, SARC, and THYM were associated with the G1/S or G2/M phase. The DNA damage checkpoint is often compromised in cancer cells, leading to sustained cell division despite accumulated genetic errors (38, 39). BUB1B and CDK1 may act in concert with HELLS, contributing to cancer malignancy and progression.

HELLS activates or inhibits several famous pathways in cancers, suggesting that HELLS regulates tumors through various mechanisms. HELLS may regulate apoptosis and cell cycle by interacting with tumor protein 53 (TP53). In hepatocellular carcinoma, TP53 inhibits HELLS, which mediates HELLS downregulation through cell cycle regulation and induces apoptosis (40). However, HELLS can act as a positive regulator in nasopharyngeal carcinoma cell lines to activate TP53 (41). P53RRA activates TP53 and the inhibition of P53RRA in human lung cancer cell lines can lead to HELLS involvement in TP53-mediated cell cycle arrest and apoptosis (42). This suggests an interplay of multiple mechanisms between HELLS and TP53 which could disrupt the delicate balance between HELLS regulation of the tumor cell cycle and apoptosis. Here, HELLS negatively correlated with apoptosis in LUAD at the single-cell level, suggesting that HELL-mediated apoptosis regulation mechanisms differ across cancers.

HELLS may also regulate cancer by mediating DNA repair and cell cycle. Damaged DNA in chromatin can be repaired by various mechanisms, including DNA methylation, posttranslational modification of histones, and nucleosome remodeling. HELLS may shift the relative orientation of two recA-like structural domains on itself by hydrolyzing ATP (43), hence increasing the accessibility of transcription factors to DNA (44) and carrying out the primary molecular biological function of reconstituting nucleosomes (45). As a key regulator of chromatin structure, HELLS helps maintain genome stability and repair DNA damage (46). Eukaryotic cells activate DDR after DNA damage and initiate two downstream pathways, canonical nonhomologous end joining (C-NHEJ) and homologous recombination (HR), to maintain genomic integrity. HELLS indirectly repairs DNA damage by positively regulating C-NHEJ and HR (47), affecting genomic homeostasis and cancer regulation (48). It recruits Ku80 and Ku70 to the location of the double-strand break, beginning at C-NHEJ (49). Interacting with ZBTB24, CDCA7, and DNMT3B, HELLS also preserves normal DNA methylation and structural repair (48, 50). In addition, H2AX is a DNA damage marker and HELLS can sustain efficient H2AX phosphorylation and DNA damage repair by ATP hydrolysis (48, 51). CtIP, a transcription factor with a C2H2 zinc finger structure, is essential for cell cycle monitoring point control and DNA damage repair. HELLS starts the HR pathway to permit homologous recombination at DNA two-ended breaks and repair heterochromatic regions in the G2 phase by boosting ATP-dependent end-resection and CtIP accumulation at DNA damage (52). In addition, HELLS catalyzes the transfer of macroH2A into mononucleosomes reconstituted by normal core histones in an ATP-dependent manner, therefore shielding nascent DNA from degradation and preserving the chromatin environment at replication forks (53–55). However, the damage is tissue-specific; although DDR operates relatively uniformly, DDR activation can vary across tissue microenvironments, which can lead to tissue-specific tumourigenesis (56). Thus, HELLS is a protective factor in some cancers but a risk factor in others. The efficacy of oncology treatments may depend on tissue-specific DDR and tumourigenesis.

Our results also showed that HELLS activates the EMT pathway in cancers and that HELLS expression positively correlates with EMT in AML. EMT enhances the migratory and invasive potential of malignant tumor cells, which may trigger anti-apoptosis and degradation of the extracellular matrix (57). Previous studies (58, 59) found that HELLS can mediate EMT in nasopharyngeal carcinoma cells by altering intermediates of tricarboxylic acid metabolism in cancer cells, promoting their migration, invasion, and progression.

Finally, we assessed the impact of HELLS on immune infiltration by analyzing its correlation with immune lymphocytes and immunomodulatory factors in the 33 cancers. DDR can affect both natural and intrinsic immunity (60); inappropriate DDR triggers abnormal immune responses in the tumor microenvironment (TME) (61, 62). Immune infiltration in the TME determines the clinical outcome of patients with cancer. Here, HELLS expression was positively and strongly correlated with Th2 cells in most tumors, whereas it was negatively or not correlated with Th1 cells. CD4+ T helper cells can differentiate into different subtypes: Th1 and Th2 cells. Th1 cells enhance the antitumor function of cytotoxic T cells *in situ* by producing several cytokines, including IL-2 and IFN-γ (63). A predominantly Th1-directed response inhibits tumor growth and increased Th1 cell infiltration is associated with a good prognosis in almost all tumors (64). In contrast, Th2 has both protumor and immunosuppressive functions and is often associated with poor prognosis in different tumors (64, 65).

Th1 and Th2 cells are balanced under normal conditions because they secrete cytokines to promote their proliferation and inhibit the proliferation of the other. But abnormal conditions can disturb this balance, as seen in cancers in which Th2 cells often dominate Th1 cells (66, 67). This phenomenon likely explains why high HELLS expression is linked to poor prognosis in many cases. The Th2/Th1 imbalance may be associated with the immune escape of tumors (65), and this, together with the association of HELLS expression with immunosuppressive markers in various cancers, suggests that HELLS can be used to identify tissue-specific programs that drive the Th2 immune response. It can also be used in conjunction with other immune, targeted, or cytotoxic therapies to block Th2 responses. HELLS expression correlates positively with Tcm cells in many cancers. Klebanoff et al. (68) found that Tcm cells show superior *in vivo* expansion, persistence, and antitumor capacity compared with effector memory T cells and effector T cells in CAR-T therapy. However, no studies have explored how patients with high HELLS expression respond to CAR-T therapy. Future clinical trials should consider using HELLS as a classifier.

In conclusion, our study elucidated the role of HELLS in pan-cancer from various angles, including its relevant signaling pathways, mutation sites, and relation to immune cell infiltration. HELLS is highly expressed in many cancers and is a potential diagnostic and prognostic marker. HELLS can activate or inhibit a variety of cancer-related pathways and is also closely associated with immune infiltration and immunomodulation. Our findings on the role of HELLS in tumor promotion and suppression are prerequisites to clinical validation and practical application of HELLS-based therapies.

## Data Availability Statement

The datasets presented in this study can be found in online repositories: https://www.jianguoyun.com/p/DWHQCkEQ1q7RChifjMQEIAA.

## Author Contributions

XL: writing the article, analysis and interpretation. LL: data mining. YF: conception and design, writing the article, critical revision of the article. All authors contributed to the article and approved the submitted version.

## Conflict of Interest

The authors declare that the research was conducted in the absence of any commercial or financial relationships that could be construed as a potential conflict of interest.

## Publisher’s Note

All claims expressed in this article are solely those of the authors and do not necessarily represent those of their affiliated organizations, or those of the publisher, the editors and the reviewers. Any product that may be evaluated in this article, or claim that may be made by its manufacturer, is not guaranteed or endorsed by the publisher.

## Supplementary Material

Supplementary Figure 1 Details of HELLS mRNA expression in tissues and cell lines. **(A)** Details of HELLS mRNA expression in different tissues, **(B)** T Details of HELLS mRNA expression in different cell lines.

Supplementary Figure 2 Correlations between HELLS and DSS in 11 cancers. DSS K-M curve for HELLS 11 cancer types. The unit of X-axis is month. **(A)** ACC, **(B)** CESC, **(C)** COAD, **(D)** KIRC, **(E)** KIRP, **(F)** LGG, **(G)** LIHC, **(H)** LUAD, **(I)** MESO, **(J)** SARC, **(K)** UCS.

Supplementary Figure 3 Correlations between HELLS and PFI in 8 cancers. PFI K-M curve for HELLS 8 cancer types. The unit of X-axis is month. **(A)** ACC, **(B)** KIRP, **(C)** LIHC, **(D)** LUAD, **(E)** MESO, **(F)** PAAD, **(G)** PRAD, **(H)** SARC.

Supplementary Figure 4 Correlations between HELLS expression and immune subtype in 14 cancers. **(A)** BRCA, **(B)** COAD, **(C)** ESCA **(D)** HNSC, **(E)** KICH, **(F)** KIRC, **(G)** OV, **(H)** PCPG, **(I)** READ, **(J)** SKCM, **(K)** STAD, **(L)** TGCT, **(M)** THCA, **(N)** UCEC.

Supplementary Figure 5 Correlations between HELLS expression and molecular subtypes in 8 cancers. **(A)** BRCA, **(B)** COAD, **(C)** HNSC, **(D)** OV, **(E)** PCPG, **(F)** SKCM, **(G)** STAD, **(H)** UCEC.

## References

[1] Basu AK Nohmi T. Chemically-Induced DNA Damage, Mutagenesis, and Cancer. Int J Mol Sci (2018) 19(6):1767–71. doi: 10.3390/ijms19061767 PMC6032311 29899224

[2] Valko M Rhodes CJ Moncol J Izakovic M Mazur M. Free Radicals, Metals and Antioxidants in Oxidative Stress-Induced Cancer. Chem Biol Interact (2006) 160(1):1–40. doi: 10.1016/j.cbi.2005.12.009 16430879

[3] Khanna KK Jackson SP. DNA Double-Strand Breaks: Signaling, Repair and the Cancer Connection. Nat Genet (2001) 27(3):247–54. doi: 10.1038/85798 11242102

[4] Jackson SP Bartek J. The DNA-Damage Response in Human Biology and Disease. Nature (2009) 461(7267):1071–8. doi: 10.1038/nature08467 PMC2906700 19847258

[5] Clapier CR Cairns BR. The Biology of Chromatin Remodeling Complexes. Annu Rev Biochem (2009) 78:273–304. doi: 10.1146/annurev.biochem.77.062706.153223 19355820

[6] Narlikar GJ Sundaramoorthy R Owen-Hughes T. Mechanisms and Functions of ATP-Dependent Chromatin-Remodeling Enzymes. Cell (2013) 154(3):490–503. doi: 10.1016/j.cell.2013.07.011 23911317 PMC3781322

[7] Rother MB van Attikum H. DNA Repair Goes Hip-Hop: SMARCA and CHD Chromatin Remodellers Join the Break Dance. Philos Trans R Soc Lond B Biol Sci (2017) 372(1731):0285–97. doi: 10.1098/rstb.2016.0285 PMC5577463 28847822

[8] Chetty R Serra S. SMARCA Family of Genes. J Clin Pathol (2020) 73(5):257–60. doi: 10.1136/jclinpath-2020-206451 32312722

[9] Zhu W Li LL Songyang Y Shi Z Li D. Identification and Validation of HELLS (Helicase, Lymphoid-Specific) and ICAM1 (Intercellular Adhesion Molecule 1) as Potential Diagnostic Biomarkers of Lung Cancer. PeerJ (2020) 8:e8731. doi: 10.7717/peerj.8731 32195055 PMC7067188

[10] Lee DW Zhang K Ning ZQ Raabe EH Tintner S Wieland R. Proliferation-Associated SNF2-Like Gene (PASG): A SNF2 Family Member Altered in Leukemia. Cancer Res (2000) 60(13):3612–22. 10910076

[11] Zeng W Baumann C Schmidtmann A Honaramooz A Tang L Bondareva A. Lymphoid-Specific Helicase (HELLS) is Essential for Meiotic Progression in Mouse Spermatocytes. Biol Reprod (2011) 84(6):1235–41. doi: 10.1095/biolreprod.110.085720 PMC3099587 21349825

[12] De La Fuente R Baumann C Fan T Schmidtmann A Dobrinski I Muegge K. Lsh is Required for Meiotic Chromosome Synapsis and Retrotransposon Silencing in Female Germ Cells. Nat Cell Biol (2006) 8(12):1448–54. doi: 10.1038/ncb1513 17115026

[13] Colak D Nofal A Albakheet A Nirmal M Jeprel H Eldali A. Age-Specific Gene Expression Signatures for Breast Tumors and Cross-Species Conserved Potential Cancer Progression Markers in Young Women. PloS One (2013) 8(5):e63204. doi: 10.1371/journal.pone.0063204 23704896 PMC3660335

[14] Ryu B Kim DS Deluca AM Alani RM. Comprehensive Expression Profiling of Tumor Cell Lines Identifies Molecular Signatures of Melanoma Progression. PloS One (2007) 2(7):e594. doi: 10.1371/journal.pone.0000594 17611626 PMC1895889

[15] Yang R Liu N Chen L Jiang Y Shi Y Mao C. GIAT4RA Functions as a Tumor Suppressor in non-Small Cell Lung Cancer by Counteracting Uchl3-Mediated Deubiquitination of LSH. Oncogene (2019) 38(46):7133–45. doi: 10.1038/s41388-019-0909-0 31417184

[16] Xiao D Huang J Pan Y Li H Fu C Mao C. Chromatin Remodeling Factor LSH is Upregulated by the LRP6-Gsk3β-E2F1 Axis Linking Reversely With Survival in Gliomas. Theranostics (2017) 7(1):132–43. doi: 10.7150/thno.17032 PMC5196891 28042322

[17] Zhang G Dong Z Prager BC Kim LJ Wu Q Gimple RC. Chromatin Remodeler HELLS Maintains Glioma Stem Cells Through E2F3 and MYC. JCI Insight (2019) 4(7):e126140–59. doi: 10.1172/jci.insight.126140 PMC6483649 30779712

[18] Gil DP Law JN Murali TM. The PathLinker App: Connect the Dots in Protein Interaction Networks. F1000Res (2017) 6:58. doi: 10.12688/f1000research.9909.1 28413614 PMC5365231

[19] Liu CJ Hu FF Xia MX Han L Zhang Q Guo AY. GSCALite: A Web Server for Gene Set Cancer Analysis. Bioinformatics (2018) 34(21):3771–2. doi: 10.1093/bioinformatics/bty411 29790900

[20] Yuan H Yan M Zhang G Liu W Deng C Liao G. CancerSEA: A Cancer Single-Cell State Atlas. Nucleic Acids Res (2019) 47(D1):D900–8. doi: 10.1093/nar/gky939 PMC6324047 30329142

[21] Waseem A Ali M Odell EW Fortune F Teh MT. Downstream Targets of FOXM1: CEP55 and HELLS are Cancer Progression Markers of Head and Neck Squamous Cell Carcinoma. Oral Oncol (2010) 46(7):536–42. doi: 10.1016/j.oraloncology.2010.03.022 20400365

[22] Janus JR Laborde RR Greenberg AJ Wang VW Wei W Trier A. Linking Expression of FOXM1, CEP55 and HELLS to Tumorigenesis in Oropharyngeal Squamous Cell Carcinoma. Laryngoscope (2011) 121(12):2598–603. doi: 10.1002/lary.22379 22109759

[23] Tao Y Liu S Briones V Geiman TM Muegge K. Treatment of Breast Cancer Cells With DNA Demethylating Agents Leads to a Release of Pol II Stalling at Genes With DNA-Hypermethylated Regions Upstream of TSS. Nucleic Acids Res (2011) 39(22):9508–20. doi: 10.1093/nar/gkr611 PMC3239205 21880597

[24] Yang X Miao BS Wei CY Dong RZ Gao PT Zhang XY. Lymphoid-Specific Helicase Promotes the Growth and Invasion of Hepatocellular Carcinoma by Transcriptional Regulation of Centromere Protein F Expression. Cancer Sci (2019) 110(7):2133–44. doi: 10.1111/cas.14037 PMC6609811 31066149

[25] Kim HE Symanowski JT Samlowski EE Gonzales J Ryu B. Quantitative Measurement of Circulating Lymphoid-Specific Helicase (HELLS) Gene Transcript: A Potential Serum Biomarker for Melanoma Metastasis. Pigment Cell Melanoma Res (2010) 23(6):845–8. doi: 10.1111/j.1755-148X.2010.00753.x PMC3192016 20727106

[26] Hou X Yang L Wang K Zhou Y Li Q Kong F. HELLS, a Chromatin Remodeler is Highly Expressed in Pancreatic Cancer and Downregulation of it Impairs Tumor Growth and Sensitizes to Cisplatin by Reexpressing the Tumor Suppressor TGFBR3. Cancer Med (2021) 10(1):350–64. doi: 10.1002/cam4.3627 PMC7826454 33280236

[27] Tang X Guo M Ding P Deng Z Ke M Yuan Y. BUB1B and Circbub1b_544aa Aggravate Multiple Myeloma Malignancy Through Evoking Chromosomal Instability. Signal Transduct Target Ther (2021) 6(1):361. doi: 10.1038/s41392-021-00746-6 34620840 PMC8497505

[28] Komura K Inamoto T Tsujino T Matsui Y Konuma T Nishimura K. Increased BUB1B/BUBR1 Expression Contributes to Aberrant DNA Repair Activity Leading to Resistance to DNA-Damaging Agents. Oncogene (2021) 40(43):6210–22. doi: 10.1038/s41388-021-02021-y PMC8553621 34545188

[29] Omori H Shan Q Takabatake K Nakano K Kawai H Sukegawa S. The Origin of Stroma Influences the Biological Characteristics of Oral Squamous Cell Carcinoma. Cancers (Basel) (2021) 13(14):3491–512. doi: 10.3390/cancers13143491 PMC8305380 34298705

[30] Vaz S Ferreira FJ Macedo JC Leor G Ben-David U Bessa J. FOXM1 Repression Increases Mitotic Death Upon Antimitotic Chemotherapy Through BMF Upregulation. Cell Death Dis (2021) 12(6):542. doi: 10.1038/s41419-021-03822-5 34035233 PMC8149823

[31] Li WH Huang K Wen FB Cui GH Guo HZ Zhao S. PLOD3 Regulates the Expression of YAP1 to Affect the Progression of non-Small Cell Lung Cancer *via* the Pkcδ/CDK1/LIMD1 Signaling Pathway. Lab Invest (2022) 102(4):440–51. doi: 10.1038/s41374-021-00674-7 35039611

[32] Malacrida A Cavaletti G Miloso M. Rigosertib and Cholangiocarcinoma: A Cell Cycle Affair. Int J Mol Sci (2021) 23(1):213–21. doi: 10.3390/ijms23010213 PMC8745771 35008638

[33] Li G Zhong Y Wang W Jia X Zhu H Jiang W. Sempervirine Mediates Autophagy and Apoptosis *via* the Akt/mTOR Signaling Pathways in Glioma Cells. Front Pharmacol (2021) 12:770667. doi: 10.3389/fphar.2021.770667 34916946 PMC8670093

[34] Cheng Q Ma Z Shi Y Parris AB Kong L Yang X. FGFR1 Overexpression Induces Cancer Cell Stemness and Enhanced Akt/Erk-ER Signaling to Promote Palbociclib Resistance in Luminal A Breast Cancer Cells. Cells (2021) 10(11):3008–29. doi: 10.3390/cells10113008 PMC8616148 34831231

[35] Yang L Dai J Ma M Mao L Si L Cui C. Identification of a Functional Polymorphism Within the 3'-Untranslated Region of Denticleless E3 Ubiquitin Protein Ligase Homolog Associated With Survival in Acral Melanoma. Eur J Cancer (2019) 118:70–81. doi: 10.1016/j.ejca.2019.06.006 31325875

[36] Liu S Gu L Wu N Song J Yan J Yang S. Overexpression of DTL Enhances Cell Motility and Promotes Tumor Metastasis in Cervical Adenocarcinoma by Inducing RAC1-JNK-FOXO1 Axis. Cell Death Dis (2021) 12(10):929. doi: 10.1038/s41419-021-04179-5 34635635 PMC8505428

[37] Zeng X Shi G He Q Zhu P. Screening and Predicted Value of Potential Biomarkers for Breast Cancer Using Bioinformatics Analysis. Sci Rep (2021) 11(1):20799. doi: 10.1038/s41598-021-00268-9 34675265 PMC8531389

[38] Huang R Zhou PK. DNA Damage Repair: Historical Perspectives, Mechanistic Pathways and Clinical Translation for Targeted Cancer Therapy. Signal Transduct Target Ther (2021) 6(1):254. doi: 10.1038/s41392-021-00648-7 34238917 PMC8266832

[39] Roos WP Thomas AD Kaina B. DNA Damage and the Balance Between Survival and Death in Cancer Biology. Nat Rev Cancer (2016) 16(1):20–33. doi: 10.1038/nrc.2015.2 26678314

[40] Schuller S Sieker J Riemenschneider P Köhler B Drucker E Weiler S. HELLS Is Negatively Regulated by Wild-Type P53 in Liver Cancer by a Mechanism Involving P21 and FOXM1. Cancers (Basel) (2022) 14(2):459–79. doi: 10.3390/cancers14020459 PMC8773711 35053620

[41] Chen L Shi Y Liu N Wang Z Yang R Yan B. DNA Methylation Modifier LSH Inhibits P53 Ubiquitination and Transactivates P53 to Promote Lipid Metabolism. Epigenet Chromatin (2019) 12(1):59. doi: 10.1186/s13072-019-0302-9 PMC6781351 31594538

[42] Mao C Wang X Liu Y Wang M Yan B Jiang Y. A G3BP1-Interacting lncRNA Promotes Ferroptosis and Apoptosis in Cancer *via* Nuclear Sequestration of P53. Cancer Res (2018) 78(13):3484–96. doi: 10.1158/0008-5472.CAN-17-3454 PMC8073197 29588351

[43] Flaus A Martin DM Barton GJ Owen-Hughes T. Identification of Multiple Distinct Snf2 Subfamilies With Conserved Structural Motifs. Nucleic Acids Res (2006) 34(10):2887–905. doi: 10.1093/nar/gkl295 PMC1474054 16738128

[44] Bartholomew B. Regulating the Chromatin Landscape: Structural and Mechanistic Perspectives. Annu Rev Biochem (2014) 83:671–96. doi: 10.1146/annurev-biochem-051810-093157 PMC4332854 24606138

[45] Ren J Briones V Barbour S Yu W Han Y Terashima M. The ATP Binding Site of the Chromatin Remodeling Homolog Lsh is Required for Nucleosome Density and *De Novo* DNA Methylation at Repeat Sequences. Nucleic Acids Res (2015) 43(3):1444–55. doi: 10.1093/nar/gku1371 PMC4330352 25578963

[46] Chen X Li Y Rubio K Deng B Li Y Tang Q. Lymphoid-Specific Helicase in Epigenetics, DNA Repair and Cancer. Br J Cancer (2022) 126(2):165–73. doi: 10.1038/s41416-021-01543-2 PMC8770686 34493821

[47] Scully R Panday A Elango R Willis NA. DNA Double-Strand Break Repair-Pathway Choice in Somatic Mammalian Cells. Nat Rev Mol Cell Biol (2019) 20(11):698–714. doi: 10.1038/s41580-019-0152-0 31263220 PMC7315405

[48] Unoki M Funabiki H Velasco G Francastel C Sasaki H. CDCA7 and HELLS Mutations Undermine Nonhomologous End Joining in Centromeric Instability Syndrome. J Clin Invest (2019) 129(1):78–92. doi: 10.1172/JCI99751 30307408 PMC6307953

[49] Basenko EY Kamei M Ji L Schmitz RJ Lewis ZA. The LSH/DDM1 Homolog MUS-30 Is Required for Genome Stability, But Not for DNA Methylation in Neurospora Crassa. PloS Genet (2016) 12(1):e1005790. doi: 10.1371/journal.pgen.1005790 26771905 PMC4714748

[50] Jenness C Giunta S Müller MM Kimura H Muir TW Funabiki H. HELLS and CDCA7 Comprise a Bipartite Nucleosome Remodeling Complex Defective in ICF Syndrome. Proc Natl Acad Sci U.S.A. (2018) 115(5):E876–85. doi: 10.1073/pnas.1717509115 PMC5798369 29339483

[51] Burrage J Termanis A Geissner A Myant K Gordon K Stancheva I. The SNF2 Family ATPase LSH Promotes Phosphorylation of H2AX and Efficient Repair of DNA Double-Strand Breaks in Mammalian Cells. J Cell Sci (2012) 125(Pt 22):5524–34. doi: 10.1242/jcs.111252 PMC3561860 22946062

[52] Kollárovič G Topping CE Shaw EP Chambers AL. The Human HELLS Chromatin Remodelling Protein Promotes End Resection to Facilitate Homologous Recombination and Contributes to DSB Repair Within Heterochromatin. Nucleic Acids Res (2020) 48(4):1872–85. doi: 10.1093/nar/gkz1146 PMC7038987 31802118

[53] Xu X Ni K He Y Ren J Sun C Liu Y. The Epigenetic Regulator LSH Maintains Fork Protection and Genomic Stability *via* MacroH2A Deposition and RAD51 Filament Formation. Nat Commun (2021) 12(1):3520. doi: 10.1038/s41467-021-23809-2 34112784 PMC8192551

[54] Ni K Muegge K. LSH Catalyzes ATP-Driven Exchange of Histone Variants Macroh2a1 and Macroh2a2. Nucleic Acids Res (2021) 49(14):8024–36. doi: 10.1093/nar/gkab588 PMC8373057 34223906

[55] Sun Z Bernstein E. Histone Variant Macroh2a: From Chromatin Deposition to Molecular Function. Essays Biochem (2019) 63(1):59–74. doi: 10.1042/EBC20180062 31015383

[56] Sun S Osterman MD Li M. Tissue Specificity of DNA Damage Response and Tumorigenesis. Cancer Biol Med (2019) 16(3):396–414. doi: 10.20892/j.issn.2095-3941.2019.0097 31565474 PMC6743622

[57] Fedele M Sgarra R Battista S Cerchia L Manfioletti G. The Epithelial-Mesenchymal Transition at the Crossroads Between Metabolism and Tumor Progression. Int J Mol Sci (2022) 23(2):800–34. doi: 10.3390/ijms23020800 PMC8776206 35054987

[58] Liu S Tao YG. Chromatin Remodeling Factor LSH Affects Fumarate Hydratase as a Cancer Driver. Chin J Cancer (2016) 35(1):72. doi: 10.1186/s40880-016-0138-7 27473869 PMC4967323

[59] He X Yan B Liu S Jia J Lai W Xin X. Chromatin Remodeling Factor LSH Drives Cancer Progression by Suppressing the Activity of Fumarate Hydratase. Cancer Res (2016) 76(19):5743–55. doi: 10.1158/0008-5472.CAN-16-0268 PMC7821962 27302170

[60] Pilger D Seymour LW Jackson SP. Interfaces Between Cellular Responses to DNA Damage and Cancer Immunotherapy. Genes Dev (2021) 35(9-10):602–18. doi: 10.1101/gad.348314.121 PMC8091970 33888558

[61] Ye Z Shi Y Lees-Miller SP Tainer JA. Function and Molecular Mechanism of the DNA Damage Response in Immunity and Cancer Immunotherapy. Front Immunol (2021) 12:797880. doi: 10.3389/fimmu.2021.797880 34970273 PMC8712645

[62] Clark CA Yang ES. Harnessing DNA Repair Defects to Augment Immune-Based Therapies in Triple-Negative Breast Cancer. Front Oncol (2021) 11:703802. doi: 10.3389/fonc.2021.703802 34631532 PMC8497895

[63] Stout RD Bottomly K. Antigen-Specific Activation of Effector Macrophages by IFN-Gamma Producing (TH1) T Cell Clones. Failure of IL-4-Producing (TH2) T Cell Clones to Activate Effector Function in Macrophages. J Immunol (1989) 142(3):760–5. 2464024

[64] Zitvogel L Kroemer G. Oncoimmunology: A Practical Guide for Cancer Immunotherapy. Gustave Roussy Cancer Center,Villejuif Cedex, France: Springer (2018). doi: 10.1007/978-3-319-62431-0.

[65] Palucka AK Coussens LM. The Basis of Oncoimmunology. Cell (2016) 164(6):1233–47. doi: 10.1016/j.cell.2016.01.049 PMC4788788 26967289

[66] Sheu BC Lin RH Lien HC Ho HN Hsu SM Huang SC. Predominant Th2/Tc2 Polarity of Tumor-Infiltrating Lymphocytes in Human Cervical Cancer. J Immunol (2001) 167(5):2972–8. doi: 10.4049/jimmunol.167.5.2972 11509647

[67] Bais AG Beckmann I Lindemans J Ewing PC Meijer CJ Snijders PJ. A Shift to a Peripheral Th2-Type Cytokine Pattern During the Carcinogenesis of Cervical Cancer Becomes Manifest in CIN III Lesions. J Clin Pathol (2005) 58(10):1096–100. doi: 10.1136/jcp.2004.025072 PMC1770745 16189158

[68] Klebanoff CA Gattinoni L Torabi-Parizi P Kerstann K Cardones AR Finkelstein SE. Central Memory Self/Tumor-Reactive CD8+ T Cells Confer Superior Antitumor Immunity Compared With Effector Memory T Cells. Proc Natl Acad Sci U.S.A. (2005) 102(27):9571–6. doi: 10.1073/pnas.0503726102 PMC1172264 15980149

