# Supplementary figures and images for: Diagnostic, Prognostic, and Immunological Roles of HELLS in Pan-Cancer: A Bioinformatics Analysis
Source: Front Immunol. 2022 Jun 14;13:870726. doi: 10.3389/fimmu.2022.870726 (PMC9237247; doi:10.3389/fimmu.2022.870726)

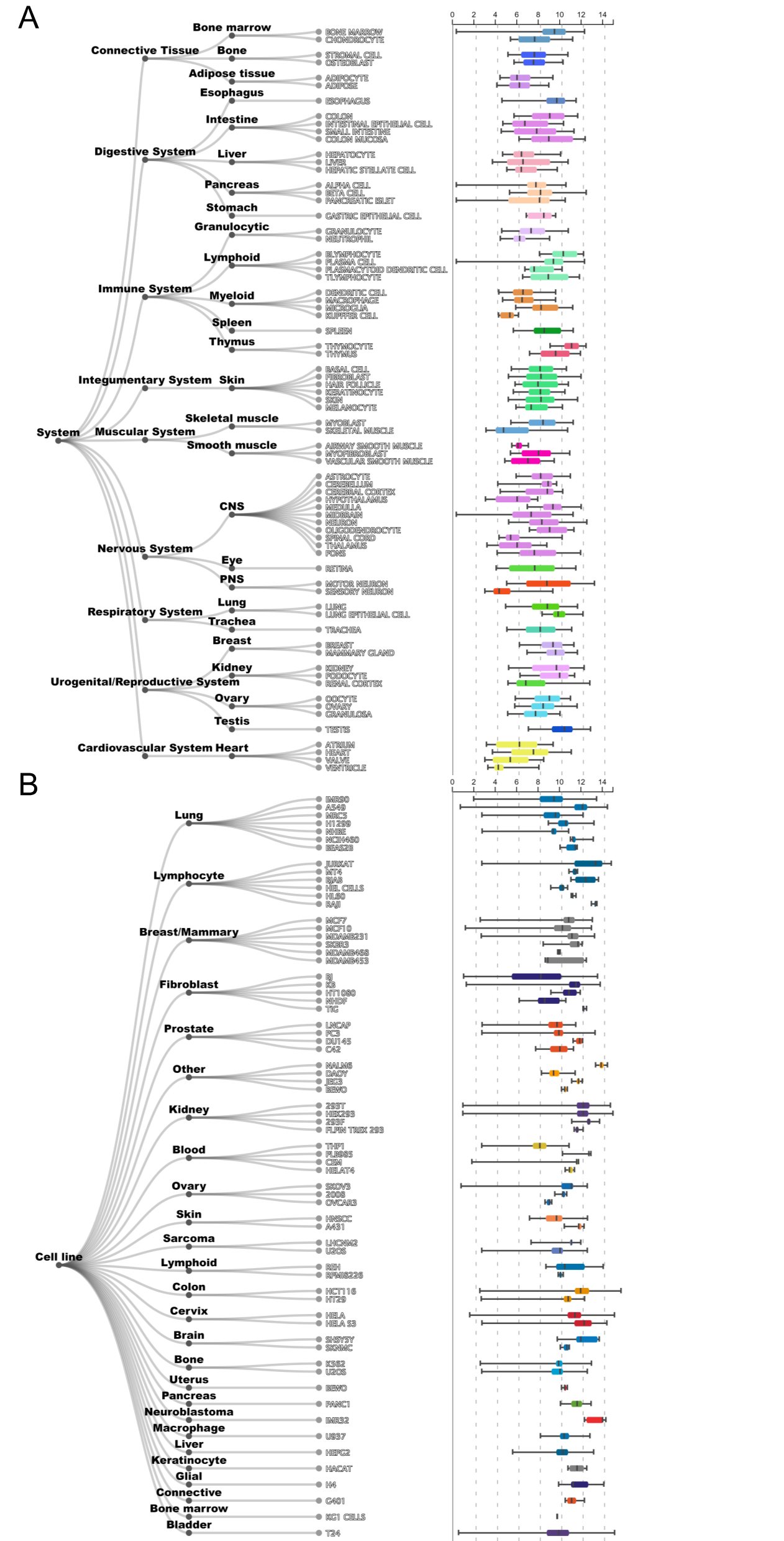

Supplement: Supplementary Figure 1 — Details of HELLS mRNA expression in tissues and cell lines. (A) Details of HELLS mRNA expression in different tissues, (B) T Details of HELLS mRNA expression in different cell lines. [file Image_1.jpeg]

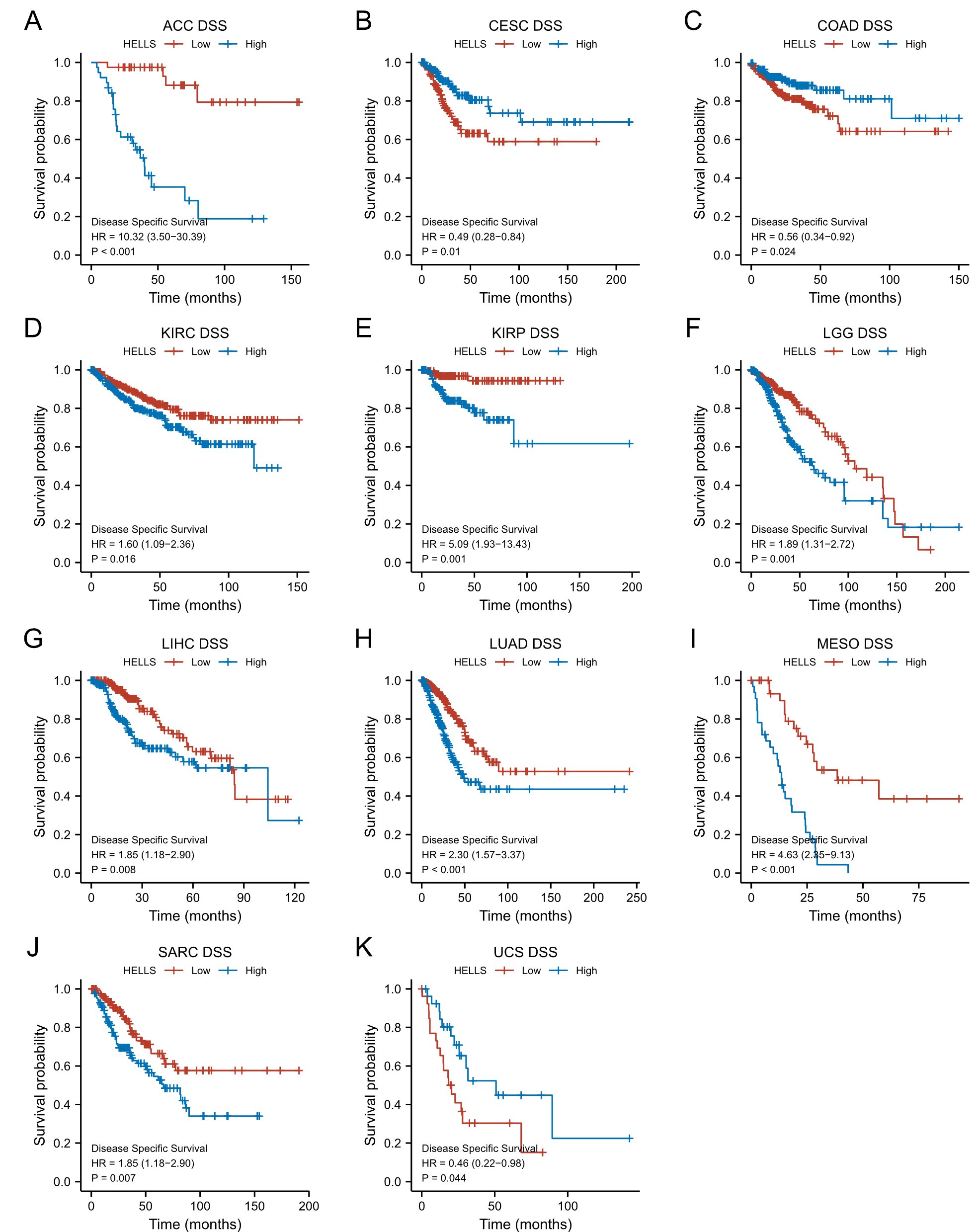

Supplement: Supplementary Figure 2 — Correlations between HELLS and DSS in 11 cancers. DSS K-M curve for HELLS 11 cancer types. The unit of X-axis is month. (A) ACC, (B) CESC, (C) COAD, (D) KIRC, (E) KIRP, (F) LGG, (G) LIHC, (H) LUAD, (I) MESO, (J) SARC, (K) UCS. [file Image_2.jpeg]

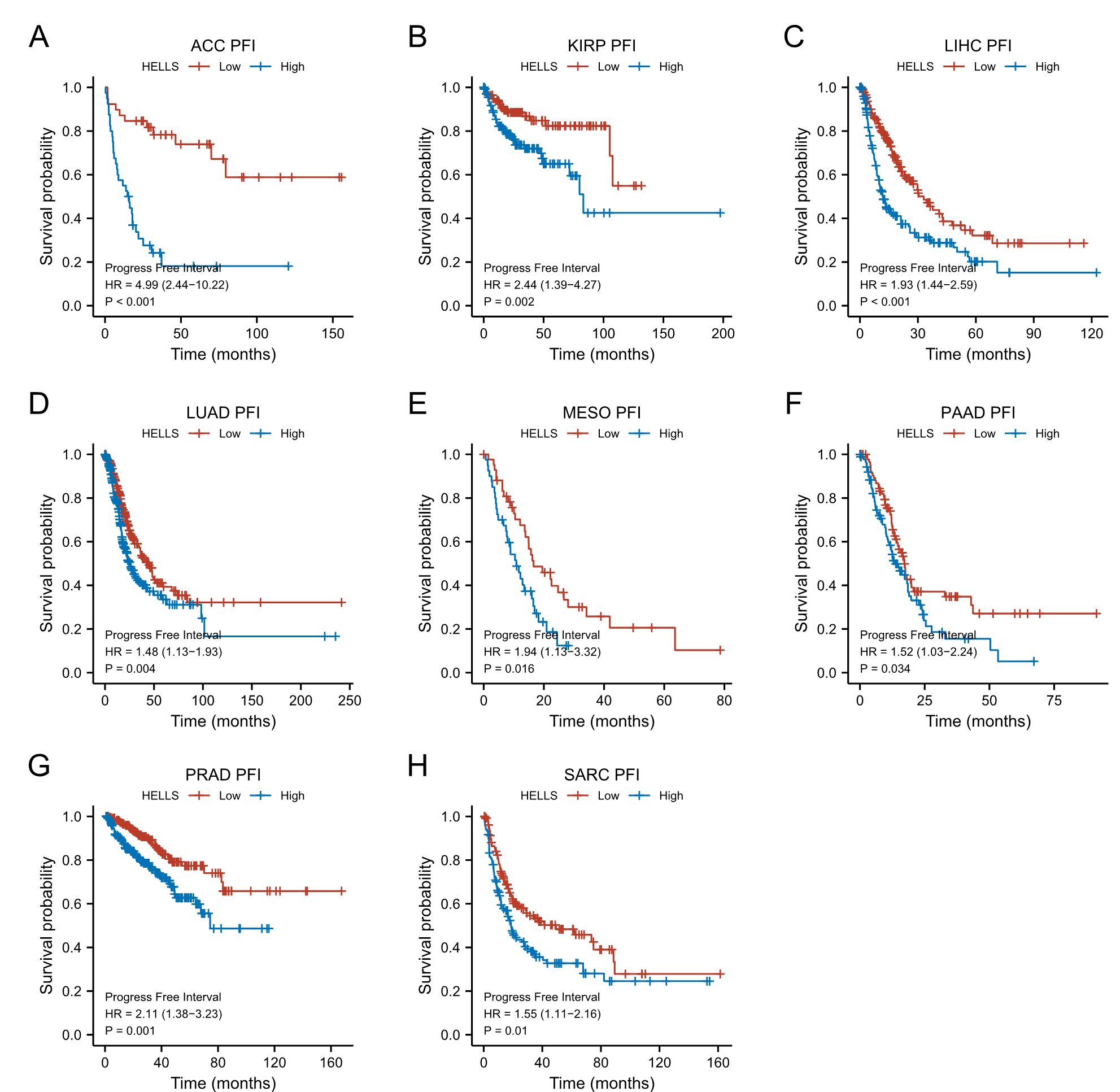

Supplement: Supplementary Figure 3 — Correlations between HELLS and PFI in 8 cancers. PFI K-M curve for HELLS 8 cancer types. The unit of X-axis is month. (A) ACC, (B) KIRP, (C) LIHC, (D) LUAD, (E) MESO, (F) PAAD, (G) PRAD, (H) SARC. [file Image_3.jpeg]

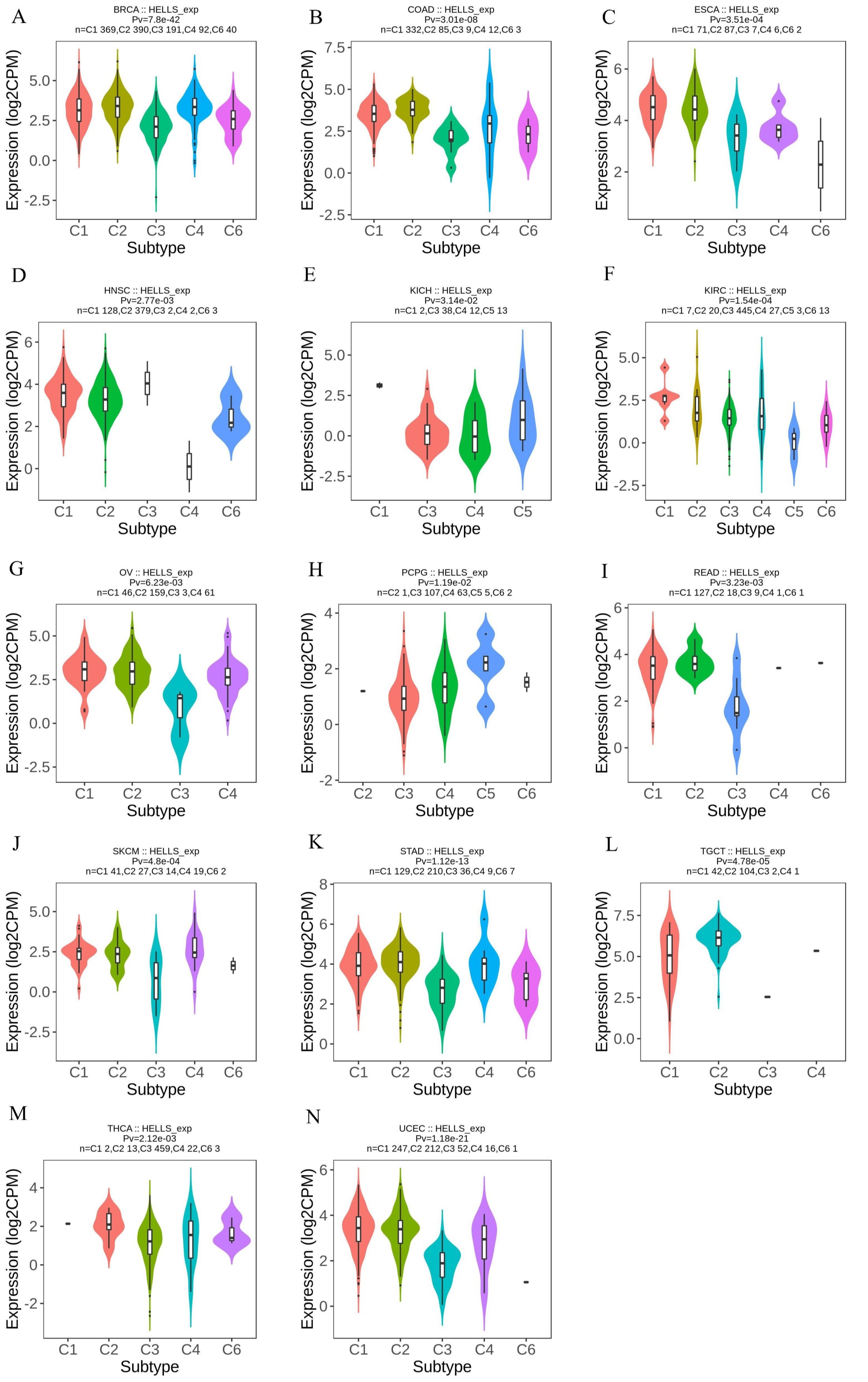

Supplement: Supplementary Figure 4 — Correlations between HELLS expression and immune subtype in 14 cancers. (A) BRCA, (B) COAD, (C) ESCA (D) HNSC, (E) KICH, (F) KIRC, (G) OV, (H) PCPG, (I) READ, (J) SKCM, (K) STAD, (L) TGCT, (M) THCA, (N) UCEC. [file Image_4.jpeg]

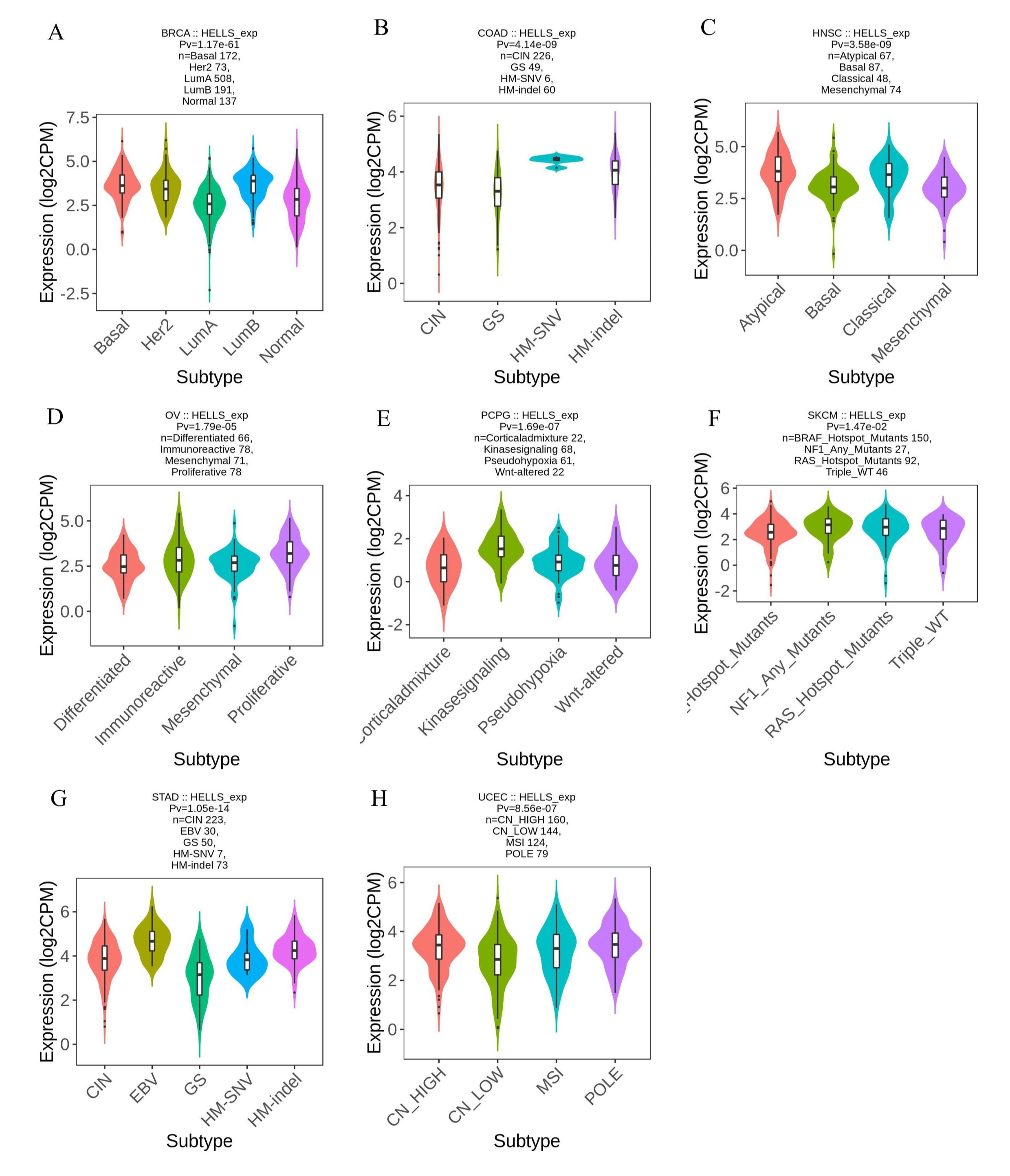

Supplement: Supplementary Figure 5 — Correlations between HELLS expression and molecular subtypes in 8 cancers. (A) BRCA, (B) COAD, (C) HNSC, (D) OV, (E) PCPG, (F) SKCM, (G) STAD, (H) UCEC. [file Image_5.jpeg]
