# Supplementary material for: Diagnostic, Prognostic, and Immunological Roles of HELLS in Pan-Cancer: A Bioinformatics Analysis
Source: Front Immunol. 2022 Jun 14;13:870726. doi: 10.3389/fimmu.2022.870726 (PMC9237247; doi:10.3389/fimmu.2022.870726)
Supplement: Supplementary file 6 [file Table_1.docx]

**Supplemental Table 1. Details of diagnostic ROC for HELLS in pan-cancer.**

| Tumor type | Tumor(n) | Normal(n) | AUC(CI) | cut-off | sensitivity | specificity | Positive predictive value | Negative predictive value | YI |
| --- | --- | --- | --- | --- | --- | --- | --- | --- | --- |
| ACC | 77 | 128 | 0.794(0.723-0.866) | 1.578 | 0.675 | 0.875 | 0.765 | 0.818 | 0.55 |
| **BLCA** | **407** | **28** | **0.921(0.882-0.929)** | **2.442** | **0.821** | **0.929** | **0.994** | **0.263** | **0.749** |
| **BRCA** | **1099** | **292** | **0.924(0.908-0.940)** | **2.159** | **0.804** | **0.897** | **0.967** | **0.549** | **0.702** |
| **CESC** | **306** | **13** | **0.993(0.984-1.000)** | **2.98** | **0.954** | **1.000** | **1.000** | **0.481** | **0.954** |
| **CHOL** | **36** | **9** | **0.997(0.998-1.000)** | **0.983** | **1.000** | **0.972** | **0.9** | **1.000** | **0.972** |
| **COAD** | **290** | **349** | **0.979(0.970-0.988)** | **2.338** | **0.928** | **0.920** | **0.906** | **0.939** | **0.847** |
| DLBC | 47 | 444 | 0.792(0.752-0.831) | 1.815 | 1.000 | 0.75 | 0.297 | 1.000 | 0.75 |
| **ESCA** | **182** | **666** | **0.983(0.975-0.991)** | **3.097** | **0.956** | **0.922** | **0.770** | **0.987** | **0.878** |
| GBM | 166 | 1157 | 0.840(0.817-0.862) | 1.183 | 0.928 | 0.758 | 0.355 | 0.987 | 0.686 |
| HNSC | 502 | 44 | 0.862(0.820-0.904) | 2.295 | 0.909 | 0.709 | 0.215 | 0.989 | 0.618 |
| KICH | 66 | 53 | 0.728(0.632-0.823) | 1.313 | 0.788 | 0.660 | 0.743 | 0.714 | 0.448 |
| KIRC | 531 | 100 | 0.585(0.516-0.653) | 1.506 | 0.725 | 0.470 | 0.879 | 0.244 | 0.195 |
| KIRP | 289 | 60 | 0.671(0.596-0.746) | 1.508 | 0.661 | 0.617 | 0.893 | 0.274 | 0.278 |
| **LAML** | **173** | **70** | **0.937(0.904-0.971)** | **3.663** | **0.896** | **0.971** | **0.987** | **0.791** | **0.867** |
| LGG | 523 | 1152 | 0.744(0.720-0.768) | 0.975 | 0.815 | 0.679 | 0.535 | 0.890 | 0.493 |
| **LIHC** | **374** | **50** | **0.942(0.913-0.971)** | **0.535** | **0.940** | **0.837** | **0.435** | **0.991** | **0.777** |
| **LUAD** | **535** | **59** | **0.960(0.944-0.977)** | **1.392** | **0.966** | **0.882** | **0.475** | **0.996** | **0.848** |
| **LUSC** | **498** | **338** | **0.969(0.958-0.980)** | **2.216** | **0.940** | **0.908** | **0.938** | **0.911** | **0.848** |
| OSCC | 329 | 32 | 0.860(0.807-0.914) | 2.295 | 0.969 | 0.650 | 0.212 | 0.995 | 0.619 |
| OV | 427 | 88 | 0.846(0.810-0.882) | 2.372 | 0.684 | 0.909 | 0.973 | 0.372 | 0.593 |
| **PAAD** | **179** | **171** | **0.979(0.967-0.992)** | **1.465** | **0.883** | **0.971** | **0.969** | **0.888** | **0.853** |
| PRAD | 496 | 152 | 0.582(0.529-0.635) | 1.43 | 0.524 | 0.612 | 0.815 | 0.283 | 0.136 |
| **READ** | **93** | **318** | **0.967(0.948-0.986)** | **2.118** | **0.935** | **0.874** | **0.685** | **0.979** | **0.810** |
| SKCM | 469 | 813 | 0.735(0.706-0.764) | 2.116 | 0.748 | 0.681 | 0.575 | 0.824 | 0.430 |
| **STAD** | **414** | **210** | **0.982(0.973-0.990)** | **2.662** | **0.930** | **0.943** | **0.970** | **0.872** | **0.873** |
| TGCT | 154 | 165 | 0.897(0.854-0.939) | 4.740 | 0.812 | 0.988 | 0.984 | 0.849 | 0.800 |
| THCA | 512 | 338 | 0.762(0.730-0.795) | 1.446 | 0.695 | 0.772 | 0.822 | 0.626 | 0.468 |
| THYM | 119 | 446 | 0.763(0.724-0.802) | 1.746 | 0.983 | 0.744 | 0.506 | 0.994 | 0.728 |
| UCEC | 552 | 35 | 0.831(0.775-0.897) | 2.445 | 0.914 | 0.697 | 0.161 | 0.992 | 0.612 |
| **UCS** | **57** | **78** | **0.945(0.910-0.980)** | **2.669** | **0.860** | **0.897** | **0.860** | **0.897** | **0.757** |

Abbreviations:ROC: Receiver Operator Characteristic curve; AUC: Area Under Curve; CI: Confidence Interval; YI: Youden’s indx.
