# Supplementary material for: Diagnostic, Prognostic, and Immunological Roles of HELLS in Pan-Cancer: A Bioinformatics Analysis
Source: Front Immunol. 2022 Jun 14;13:870726. doi: 10.3389/fimmu.2022.870726 (PMC9237247; doi:10.3389/fimmu.2022.870726)
Supplement: Supplementary file 7 [file Table_2.docx]

**Supplemental Table 2. Details of the prognostic K-M analysis of HELLS in pan-cancer.**

|  | **Cancer** | **N** | **HR (95% CI)** | **P value** |
| --- | --- | --- | --- | --- |
| **OS** |  |  |  |  |
|  | ACC | 79 | 10.71(3.67-31.28) | <0.001 |
|  | BLCA | 433 | 1.03(0.77-1.38) | 0.837 |
|  | BRCA | 1222 | 1.16(0.85-1.60) | 0.353 |
|  | CESC | 309 | 0.53(0.33-0.85) | 0.009 |
|  | CHOL | 45 | 1.09(0.43-2.77) | 0.857 |
|  | COAD | 521 | 0.74(0.50-1.10) | 0.137 |
|  | DLBC | 48 | 0.78(0.18-3.38) | 0.742 |
|  | ESCA | 173 | 1.17(0.71-1.92) | 0.548 |
|  | GBM | 174 | 1.14(0.81-1.61) | 0.458 |
|  | HNSC | 546 | 1.02(0.78-1.33) | 0.91 |
|  | KICH | 89 | 2.15(0.54-8.60) | 0.281 |
|  | KIRC | 611 | 1.15(0.86-1.56) | 0.345 |
|  | KIRP | 321 | 3.80(1.90-4.60) | <0.001 |
|  | LAML | 151 | 1.22(0.80-1.86) | 0.365 |
|  | LGG | 529 | 1.87(1.32-2.64) | <0.001 |
|  | LIHC | 424 | 1.54(1.09-2.18) | 0.015 |
|  | LUAD | 594 | 1.59(1.19-2.12) | 0.002 |
|  | LUSC | 551 | 0.71(0.54-0.93) | 0.012 |
|  | MESO | 86 | 3.42(2.04-5.73) | <0.001 |
|  | OSCC | 361 | 1.29(0.93-1.78) | 0.13 |
|  | OV | 379 | 0.94(0.72-1.21) | 0.629 |
|  | PAAD | 182 | 1.57(1.04-2.38) | 0.032 |
|  | PCPG | 186 | 1.73(0.41-7.25) | 0.456 |
|  | PRAD | 551 | 10.57(1.29-86.43) | 0.028 |
|  | READ | 177 | 0.72(0.33-1.56) | 0.406 |
|  | SKCM | 472 | 1.17(0.89_1.53) | 0.256 |
|  | SARC | 265 | 1.88(1.25-2.84) | 0.002 |
|  | STAD | 407 | 0.89(0.64-1.23) | 0.466 |
|  | TGCT | 156 | 2.92(0.30-28.03) | 0.354 |
|  | THCA | 568 | 0.61(0.22-1.68) | 0.341 |
|  | THYM | 121 | 0.16(0.03-0.84) | 0.031 |
|  | UCEC | 587 | 0.79(0.53-1.19) | 0.264 |
|  | UCS | 56 | 0.52(0.26-1.05) | 0.07 |
|  | UVM | 80 | 0.99(0.43-2.30) | 0.988 |
|  |  |  |  |  |
| **DSS** |  |  |  |  |
|  | ACC | 79 | 10.32(3.5-30.39) | <0.001 |
|  | BLCA | 433 | 1.11(0.78-1.58) | 0.568 |
|  | BRCA | 1222 | 1.07(0.7-1.64) | 0.758 |
|  | CESC | 309 | 0.49(0.28-0.84) | 0.01 |
|  | CHOL | 45 | 0.85(0.31-2.32) | 0.756 |
|  | COAD | 521 | 0.56(0.34-0.92) | 0.024 |
|  | DLBC | 48 | 1.32(0.18-9.64) | 0.768 |
|  | ESCA | 173 | 1.33(0.74-2.39) | 0.342 |
|  | GBM | 174 | 1.20(0.83-1.73) | 0.339 |
|  | HNSC | 546 | 1.06(0.75-1.49) | 0.755 |
|  | KICH | 89 | 6.52(0.78-54.28) | 0.083 |
|  | KIRC | 611 | 1.60(1.09-2.36) | 0.016 |
|  | KIRP | 321 | 5.09(1.93-13.43) | 0.001 |
|  | LAML | 151 |  |  |
|  | LGG | 529 | 1.89(1.31-2.72) | 0.001 |
|  | LIHC | 424 | 1.85(1.18-2.90) | 0.008 |
|  | LUAD | 594 | 2.30(1.57-3.37) | <0.001 |
|  | LUSC | 551 | 0.86(0.57-1.32) | 0.498 |
|  | MESO | 86 | 4.63(2.35-9.13) | <0.001 |
|  | OSCC | 361 | 1.25(0.83-1.87) | 0.291 |
|  | OV | 379 | 0.91(0.69-1.20) | 0.509 |
|  | PAAD | 182 | 1.45(0.91-2.30) | 0.119 |
|  | PCPG | 186 | 2.12(0.39-11.63) | 0.387 |
|  | PRAD | 551 | inf(0-inf) | 0.999 |
|  | READ | 177 | 0.85(0.30-2.43) | 0.757 |
|  | SKCM | 472 | 1.18(0.88-1.57) | 0.26 |
|  | SARC | 265 | 1.85(1.18-2.90) | 0.007 |
|  | STAD | 407 | 1.00(0.66-1.52) | 0.993 |
|  | TGCT | 156 | 1.92(0.17-21.23) | 0.593 |
|  | THCA | 568 |  |  |
|  | THYM | 121 | 0.13(0.01-1.50) | 0.102 |
|  | UCEC | 587 | 0.74(0.45-1.22) | 0.238 |
|  | UCS | 56 | 0.46(0.22-0.98) | 0.044 |
|  | UVM | 80 | 0.87(0.36-2.07) | 0.747 |
|  |  |  |  |  |
| **PFI** |  |  |  |  |
|  | ACC | 79 | 4.99(2.44-10.22) | <0.001 |
|  | BLCA | 433 | 1.15(0.85-1.54) | 0.362 |
|  | BRCA | 1222 | 0.87(0.63-1.20) | 0.383 |
|  | CESC | 309 | 0.70(0.11-1.11) | 0.132 |
|  | CHOL | 45 | 0.80(0.33-1.97) | 0.628 |
|  | COAD | 521 | 0.76(0.53-1.07) | 0.115 |
|  | DLBC | 48 | 1.66(0.48-5.71) | 0.422 |
|  | ESCA | 173 | 1.03(0.66-1.60) | 0.908 |
|  | GBM | 174 | 0.95(0.67-1.33) | 0.748 |
|  | HNSC | 546 | 1.15(0.87-1.53) | 0.332 |
|  | KICH | 89 | 3.10(0.83-11.72) | 0.095 |
|  | KIRC | 611 | 1.17(0.85-1.59) | 0.336 |
|  | KIRP | 321 | 2.44(1.39-4.27) | 0.002 |
|  | LAML | 151 |  |  |
|  | LGG | 529 | 1.30(0.99-1.70) | 0.062 |
|  | LIHC | 424 | 1.93(1.44-2.59) | <0.001 |
|  | LUAD | 594 | 1.48(1.13-1.93) | 0.004 |
|  | LUSC | 551 | 0.93(0.67-1.28) | 0.653 |
|  | MESO | 86 | 1.94(1.13-3.32) | 0.016 |
|  | OSCC | 361 | 1.08(0.77-1.52) | 0.638 |
|  | OV | 379 | 0.80(0.63-1.02) | 0.071 |
|  | PAAD | 182 | 1.52(1.03-2.24) | 0.034 |
|  | PCPG | 186 | 1.55(0.66-3.63) | 0.316 |
|  | PRAD | 551 | 2.11(1.38-3.23) | 0.001 |
|  | READ | 177 | 0.85(0.44-1.62) | 0.618 |
|  | SKCM | 472 | 1.18(0.94-1.46) | 0.146 |
|  | SARC | 265 | 1.55(1.11-2.16) | 0.01 |
|  | STAD | 407 | 1.03(0.73-1.47) | 0.854 |
|  | TGCT | 156 | 1.18(0.63-2.21) | 0.596 |
|  | THCA | 568 | 1.55(0.90-2.67) | 0.112 |
|  | THYM | 121 | 0.69(0.28-1.71) | 0.426 |
|  | UCEC | 587 | 0.91(0.65-1.29) | 0.603 |
|  | UCS | 56 | 0.61(0.31-1.19) | 0.147 |
|  | UVM | 80 | 1.61(0.74-3.50) | 0.228 |
