# Supplementary material for: Diagnostic, Prognostic, and Immunological Roles of HELLS in Pan-Cancer: A Bioinformatics Analysis
Source: Front Immunol. 2022 Jun 14;13:870726. doi: 10.3389/fimmu.2022.870726 (PMC9237247; doi:10.3389/fimmu.2022.870726)
Supplement: Supplementary file 8 [file Table_3.docx]

**Supplemental TABLE 3. GO and KEGG enrichment analysis for** **genes closely interact to HELLS expression.**

| ONTOLOGY | ID | Description | GeneRatio | BgRatio | pvalue | p.adjust | qvalue |
| --- | --- | --- | --- | --- | --- | --- | --- |
| BP | GO:0006260 | DNA replication | 20/50 | 274/18670 | 3.40e-24 | 3.71e-21 | 2.46e-21 |
| BP | GO:0000819 | sister chromatid segregation | 13/50 | 189/18670 | 1.98e-15 | 1.08e-12 | 7.17e-13 |
| BP | GO:0071103 | DNA conformation change | 15/50 | 327/18670 | 4.21e-15 | 1.33e-12 | 8.81e-13 |
| BP | GO:0000280 | nuclear division | 16/50 | 407/18670 | 4.86e-15 | 1.33e-12 | 8.81e-13 |
| BP | GO:0048285 | organelle fission | 16/50 | 449/18670 | 2.24e-14 | 4.88e-12 | 3.24e-12 |
| CC | GO:0098687 | chromosomal region | 19/50 | 349/19717 | 5.83e-21 | 6.36e-19 | 2.82e-19 |
| CC | GO:0000775 | chromosome, centromeric region | 11/50 | 193/19717 | 1.59e-12 | 8.67e-11 | 3.85e-11 |
| CC | GO:0042555 | MCM complex | 5/50 | 12/19717 | 6.67e-11 | 2.42e-09 | 1.08e-09 |
| CC | GO:0000793 | condensed chromosome | 10/50 | 223/19717 | 1.94e-10 | 5.28e-09 | 2.34e-09 |
| CC | GO:0005819 | spindle | 11/50 | 347/19717 | 8.67e-10 | 1.89e-08 | 8.39e-09 |
| MF | GO:0003688 | DNA replication origin binding | 7/49 | 24/17697 | 2.66e-13 | 2.81e-11 | 1.91e-11 |
| MF | GO:0140097 | catalytic activity, acting on DNA | 12/49 | 213/17697 | 4.23e-13 | 2.81e-11 | 1.91e-11 |
| MF | GO:0003697 | single-stranded DNA binding | 9/49 | 113/17697 | 2.12e-11 | 9.39e-10 | 6.39e-10 |
| MF | GO:0003678 | DNA helicase activity | 7/49 | 81/17697 | 2.38e-09 | 7.90e-08 | 5.38e-08 |
| MF | GO:0004386 | helicase activity | 8/49 | 163/17697 | 1.43e-08 | 3.79e-07 | 2.58e-07 |
| KEGG | hsa04110 | Cell cycle | 14/26 | 124/8076 | 1.58e-19 | 9.49e-18 | 7.83e-18 |
| KEGG | hsa03030 | DNA replication | 5/26 | 36/8076 | 8.11e-08 | 2.43e-06 | 2.01e-06 |
| KEGG | hsa05203 | Viral carcinogenesis | 5/26 | 204/8076 | 4.18e-04 | 0.007 | 0.006 |
| KEGG | hsa00270 | Cysteine and methionine metabolism | 3/26 | 50/8076 | 5.25e-04 | 0.007 | 0.006 |
| KEGG | hsa05166 | Human T-cell leukemia virus 1 infection | 5/26 | 219/8076 | 5.78e-04 | 0.007 | 0.006 |
